# Visualizing Clinical Evidence: Citation Networks for the Incubation Periods of Respiratory Viral Infections

**DOI:** 10.1371/journal.pone.0019496

**Published:** 2011-04-29

**Authors:** Nicholas G. Reich, Trish M. Perl, Derek A. T. Cummings, Justin Lessler

**Affiliations:** 1 Department of Epidemiology, Johns Hopkins Bloomberg School of Public Health, Baltimore, Maryland, United States of America; 2 Division of Infectious Diseases, Johns Hopkins University School of Medicine, Baltimore, Maryland, United States of America; University of Hong Kong, Hong Kong

## Abstract

Simply by repetition, medical facts can become enshrined as truth even when there is little empirical evidence supporting them. We present an intuitive and clear visual design for tracking the citation history of a particular scientific fact over time. We apply this method to data from a previously published literature review on the incubation period of nine respiratory viral infections. The resulting citation networks reveal that the conventional wisdom about the incubation period for these diseases was based on a small fraction of available data and in one case, on no retrievable empirical evidence. Overall, 50% of all incubation period statements did not provide a source for their estimate and 65% of original sources for incubation period data were not incorporated into subsequent publications. More standardized and widely available methods for visualizing these histories of medical evidence are needed to ensure that conventional wisdom cannot stray too far from empirically supported knowledge.

## Introduction

Repeated scientific and medical facts often gain a currency that far exceeds that which is warranted by the evidence that supports them. A classic example is the often-repeated recommendation that we need to drink at least eight glasses of water a day. While widely accepted as fact for years, a review of the literature showed that this recommendation was not supported by any particular piece of scientific evidence [1]. In this manuscript, we present visualizations of citation networks for particular medical facts, the incubation periods of respiratory viral infections. Citation networks are an informative way to visualize the scientific history and dissemination of specific scientific facts.

The incubation period, the time between infection with a pathogen and the onset of symptoms, is an important natural history parameter of disease. Estimates of the full distribution of the incubation period for a disease can provide practical guidance to clinicians or policy-makers in a variety of settings. For example, the incubation period may be used when determining the source of illness [2], [3] or when setting social-distancing measures such as quarantine or school-closure in an infectious disease outbreak setting [4], [5]. However, finding precise data on incubation periods is difficult because the events that define an incubation period — infection and symptom onset — may themselves be hard to observe.

Because of the challenges with collecting complete incubation period data, statements of the incubation period based on no empirical data or anecdotal data are common. Until recently, this was the state of the knowledge of the incubation periods for most common respiratory viral pathogens. To address this gap in the scientific literature, we published a systematic review of the incubation period of nine common respiratory viral infections [6]. This review provided evidence-based estimates of the full incubation period distribution for each of these diseases, incorporating data from primary sources identified through the review. In the process of reviewing 556 articles, we constructed networks that represent the history and development of knowledge about the incubation period for these diseases. The full bibliography is available in References S1.

This paper presents citation networks created from the literature review's citation data. While the trees are interesting themselves from a data visualization perspective, they also give insight into how scientific knowledge of particular medical facts evolve over time.

## Methods

A systematic literature review, described in Lessler et al. [6], was performed to identify statements of incubation period and sources of original incubation period data for the following respiratory viral infections: adenovirus, human coronavirus (HCoV), SARS-associated coronavirus, influenza A and B, measles virus, human metapneumovirus, parainfluenza, respiratory syncytial virus (RSV), and rhinovirus. All citations for a given statement were followed to identify the original source of the estimate. For each statement, all citations were recorded. These citations, an instance of one source citing another, make up the branches of the citation trees; the statements and sources are the nodes. Sources that provided no citation and were not themselves cited by another source were only included in the networks if they contained original data.

A spreadsheet was used to create a record of all statements of incubation period, whether a citation was provided or not, and all sources of data. A version of this spreadsheet is available in Data S1. This information was used to create the graphs, by hand, using Adobe Illustrator.

To visually clarify the presented citations, we incorporated a few design elements. Each citation is designated by two lines of text and an “incubation period clock”. The clocks encode the incubation period estimate visually, allowing readers to quickly grasp how the estimates change (or not) over time. Arrows indicate a precise statement (median or mean) of the incubation period while shaded blue areas indicate given ranges or confidence intervals. The top line of text succinctly summarizes the stated estimate from that source, using “h” and “d” as abbreviations for “hours” and “days”, respectively. An asterisk indicates that the source did not provide a stated incubation period, but was included because it did provide original data that could be used to estimate the incubation period. Citations shown in red or orange text are those with original data and those in black are sources that do not contain original data. Also on the first line, the abbreviation “Obs” or “Exp” appears, indicating whether the study was observational (in orange text) or experimental (in red). The second line of text gives the last name of the first author and the year in which the source was first published. The weight or stroke of the arrow lines pointing to a given source corresponds to the cumulative number of citations that rely on that source for information. We used lightly shaded gray boxes to group multiple editions of the same source and multiple publications from the same study (for example, yearly CDC influenza reports). In the case of influenza the time scale is log transformed (with 1890 as year 1) to accommodate the greater frequency of citations in later years. In all other cases the time axis follows a linear scale.

All citation trees were created as described above except for the case of SARS. The literature on SARS had markedly different characteristics from the other diseases. There is a dense and highly-connected citation network for SARS which developed over the course of the few years after the 2002 SARS outbreak. Different methods were required to create this citation network and it is available as Figure S1 for comparison purposes.

Additionally, metrics that characterize the connectedness of each network were computed to provide additional insight into the structure of the networks and direct comparisons between diseases. Based on the methods of Kleinberg [7], hub and authority scores were computed. Hub and authority scores are values between 0 and 1, computed so that the vector norm of each set of scores is equal to 1. A high hub score value for a particular source indicates that the given paper provides links to many strong authorities on the topic. A high authority score for a particular source indicates that the given paper is cited by many other sources. The results of these calculations and a complete description of the metrics calculated are given in Appendix S1.

## Results

**Figure 1:**
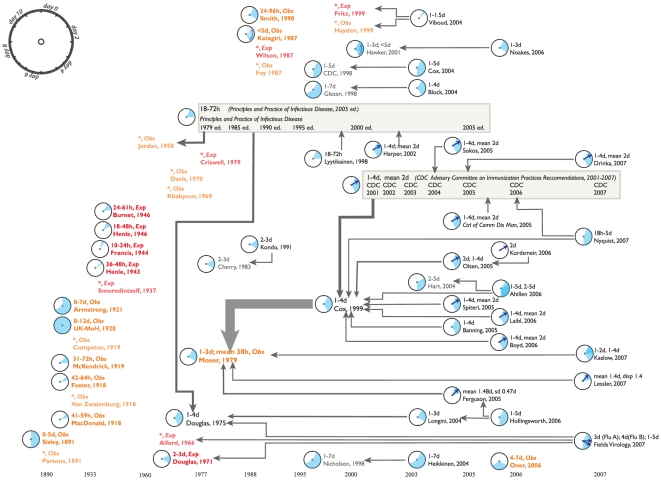
A citation network for the incubation period of influenza A and B. The most cited paper (Cox, 1999) is in the middle of the figure, while the original data that is most often relied upon (Moser, 1979) has the heaviest arrow pointing towards it. Many sources of original data are not cited at all.

We present short disease-specific discussions of our findings, illustrated by Figures 1 through [Fig pone-0019496-g002] [Fig pone-0019496-g003] 4, and summarize the metrics characterizing the networks.

**Figure 2 pone-0019496-g002:**
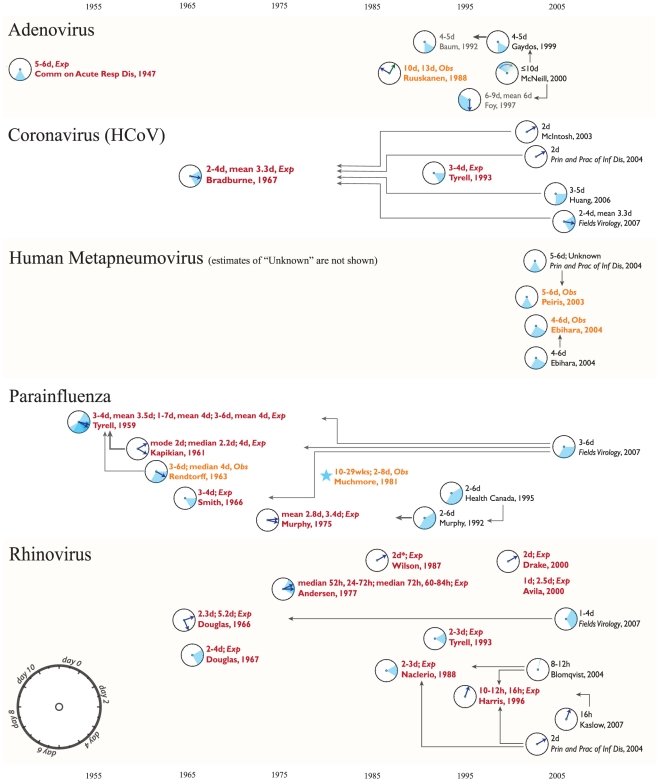
Citation networks for the incubation period of adenovirus, human coronavirus, human metapneumovirus, parainfluenza and rhinovirus.

**Figure 3 pone-0019496-g003:**
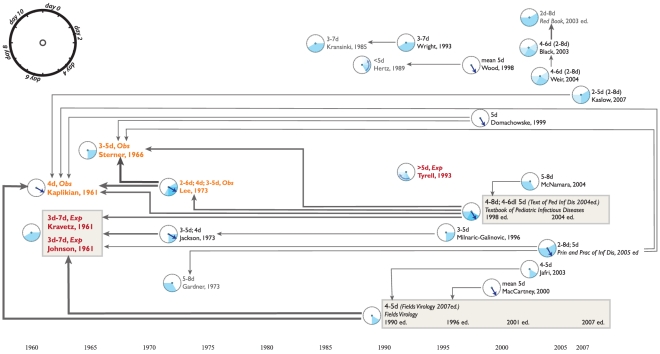
A citation network for the incubation period of respiratory syncytial virus.

**Figure 4:**
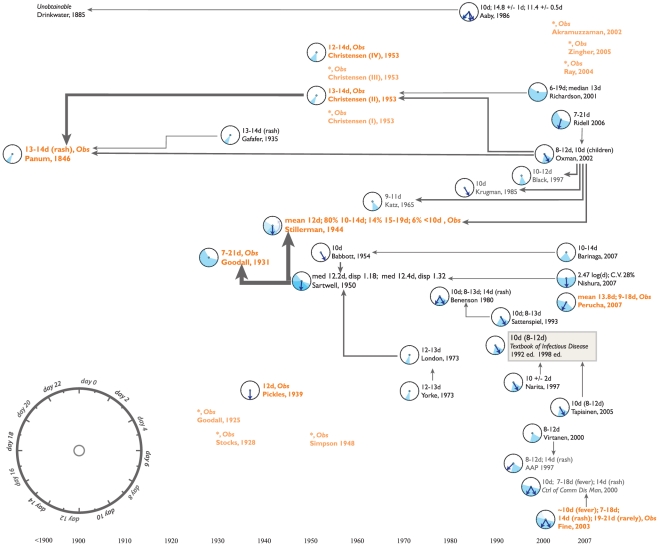
A citation network for the incubation period of measles.

### Influenza

Figure 1 captures a long history of incubation period data collection for both influenza A and B. However, as the citation network shows, only 6 of 28 (21.4%) of the original sources of data have contributed to the conventional scientific wisdom about influenza. Cox and Subbarao's claim (in 1999) that the incubation period of influenza is “1–4 days” is the single most cited statement of the incubation period of influenza [8]. Cox and Subbarao in turn cite the Moser (1979) analysis of exposure and onset times for 37 influenza cases as an example of “a single infected person [transmitting] the virus to a large number of susceptible individuals.” While this is the only possible source given by the authors for the incubation period itself, it is unclear to what extent they relied on the results in Moser et al. to inform their statement of this fact. The longest observed incubation period reported by Moser et al. was 72 hours [9]. Over 150 other usable observations on the incubation period, across 6 papers, are not cited at all as sources for influenza incubation period data.

### Adenovirus

The example of adenovirus shows that the cited incubation period estimates in some cases are not based on experimental data at all. Neither of the two original sources of data are used to inform stated estimates of the incubation period (see Figure 2).

### Coronavirus

One of two original sources of data for the incubation period for HCoV is used by subsequent authors. While one experimental study is overlooked, Figure 2 shows that the other source clearly has become the canonical source of incubation period data for HCoV. However, the interpretations of the used data are inconsistent. One of the citing sources claims the incubation period is 3–5 days, another 2–4 days and two sources identify 2 days as the incubation period. All of these reduce the information contained in the original estimate, which provides a mean as well as a range.

### Human metapneumovirus

Both original sources of data on the incubation period of human metapneumovirus are cited by subsequent authors. With just four sources on the citation network for human metapneumovirus (shown in Figure 2), the original sources are identified and interpreted correctly by subsequent authors.

### Parainfluenza

Four of six sources of original incubation period data for parainfluenza are cited by outside sources (see Figure 2). As with Rhinovirus, there are more sources of original data than there are referencing articles.

### Rhinovirus

Only three of the nine original sources providing incubation period data on rhinovirus are used by subsequent authors, as is seen in Figure 2. Several of the more recent studies, published in 2000, have not been incorporated into the accepted literature.

### Respiratory syncytial virus

As Figure 3 shows, five of six original data sources on the incubation period of respiratory syncytial virus are used by subsequent authors. The single paper that is not cited (Tyrell, 1993) provides data on RSV, rhinovirus and HCoV but is not cited subsequently in literature for any of these diseases. The RSV citations are fairly centralized around several key primary and secondary sources, although there are a few stray references that cannot be traced back to an original data source.

### Measles

Only four of 16 original sources of information on the incubation period of measles are cited, although five of those have been published since 2001. While this shows that the measles citation network does not use the available information as efficiently, Figure 4 reveals similar patterns to the citation network for RSV. Several key primary sources serve as hubs in the citation network, while a few references cannot be traced back to original data.

### Quantitative comparison of the networks

Results from our quantitative analysis of the networks are available in Appendix S1.

Influenza and human coronavirus are the two diseases with high maximum authority scores (0.99 and 1.00, respectively). These reflect the fact that the prevailing wisdom about the incubation period for these two diseases relies highly on a single source. No other disease has a maximum authority score above 0.75. Measles has the lowest maximum authority score (0.46), indicating that no one source dominates the citation network.

Adenovirus and measles have the two highest maximum hub scores (1.00 and 0.95 respectively). Influenza has the lowest maximum hub score (0.29). Maximum hub scores vary widely across diseases. However we observed that sources with high hub scores are often not highly cited. Hence, in the absence of a widely cited review, it is unclear how the presence of a paper with a high hub score influences the evolution of knowledge about the incubation period of a particular disease. For example, Oxman (2002) cites six papers in stating an estimate for the incubation period of measles, however, as of the time when the review was conducted, this paper had only been cited once.

## Discussion

The incubation period is commonly used to identify nosocomial infections and to pinpoint the origin of a single-source outbreak. The use of faulty data in these contexts can lead to inappropriate conclusions. For instance, if a clinician relied upon the incubation period listed in the 2004 edition of the *Textbook of Pediatric Infectious Diseases* for RSV (between 4 and 8 days) they would falsely conclude that a patient who developed symptoms after 3 days in a hospital had no chance of being nosocomially infected. However our best estimates for the incubation period of RSV indicates that 1 out of 3 patients with RSV will have incubation periods less than 4 days [6].

For all of the diseases presented here, we reveal that the conventional wisdom about the incubation period is often not based on much actual experimental data. Overall (excluding SARS), 35% (25/71) of original sources for incubation period data were cited by subsequent publications and 50% of all incubation period statements did not provide a source for their estimate [6]. For all diseases except for human metapneumovirus there was at least one study with original data that was not cited by any of the subsequent papers. These types of summary measures, a qualitative sense of which can be gleaned from a quick look at the networks themselves, are helpful in evaluating the need for further work in a given area.

Social factors may influence downstream citation patterns. For example, papers written by well-known authors or in high-impact journals may have a wider and faster circulation than others. Also, the accessibility of the publication (i.e. subscription required or available for free online) may also have an impact on how widely a particular publication is read and cited. Comprehensive reviews should ensure that all sources of data are brought to light, and help provide equal footing for publications that might otherwise receive less attention.

It is important to note that a complete systematic review requires more than just evaluating the use or disuse of available data. A full review of an area of knowledge requires a detailed examination of data sources and their ability to answer the question of interest. While striving to create an authoritative source on a particular topic, reviewers must strike a balance between quality and diversity of data. Including all data (regardless of quality) can lead to biased or highly variable estimates because of differing procedures for measuring data like possible exposures or time of onset. However, leaving out particular datasets because of perceived poor quality could lead to a more homogenous dataset that ignores variation due to characteristics that may change drastically from one dataset to another, like population demographics or different strains of a disease. Given all of these potential complications, a systematic review process, such as those recommended by the Cochrane Collaboration, is vital to conducting standardized, reproducible research [10].

Using enhanced data visualization techniques to display literature review data can reveal historical citation patterns and help communicate the results quickly and intuitively to a wide audience. The visualization process can be rewarding, but it is also a time-consuming and challenging task to create accurate, detailed and visually pleasing figures. Once created, however, such visual displays of citation histories can be very valuable for learning about the history and development of a particular field of research.

Research and development of software that could automate the process of creating (and easily updating) customized citation networks would be a welcome addition to the field of data visualization and any scientific realm in which comprehensive literature reviews are conducted. If it were standard practice to maintain such “evidential trees” for scientific and medical facts it would be easier to assess the weight which these facts should actually be given in practice and to determine when systematic reviews are necessary.

## Supporting Information

Appendix S1 Detailed methodology on and results from computing Kleinberg's network metrics.
(PDF)

Data S1 Raw data used to build the citation networks. Each row represents a citation and includes the incubation period estimate as stated by the citing source. The “ID” column may be cross-referenced with References S1 for complete bibliographic information.
(XLS)

Figure S1 **A citation network for the incubation period of SARS.** Because SARS is a new pathogen, the chronology of the literature review was harder to display accurately than it was for the other diseases. All papers discussing the incubation period of SARS were published in a narrow window of time between 2002 and 2007. SARS also had the most statements (171) of any of the diseases we examined. We made a more space-efficient citation network of the SARS sources using the software Pajek (http://pajek.imfm.si/doku.php). In this network, the size of the node is proportional to the total number of sources cited by a paper plus the number of sources that cite that paper. Orange nodes indicate original sources. Red nodes indicate a source with a provided citation. Blue nodes are sources that are cited but do not have original data or a citation. Arrows point to a source from the article that cited it. Observational studies are represented by boxes and experimental studies are represented by ellipses.
(EPS)

References S1 A complete bibliography of the sources found by the literature review.
(PDF)
