# Supplementary material for: Visualizing Clinical Evidence: Citation Networks for the Incubation Periods of Respiratory Viral Infections
Source: PLoS One. 2011 Apr 29;6(4):e19496. doi: 10.1371/journal.pone.0019496 (PMC3084881; doi:10.1371/journal.pone.0019496)
Supplement: Appendix S1 — Detailed methodology on and results from computing Kleinberg's network metrics. (PDF) [file pone.0019496.s001.pdf]

Visualizing clinical evidence: citation networks for the incubation periods of respiratory viral infections
Appendix S1: measurements of network characteristics

Nicholas G. Reich, Trish M. Perl, Derek A. T. Cummings, Justin Lessler

We computed the following metrics to characterize the connectedness of each citation network: "hub scores" and "authority scores" based on the methods of Kleinberg (1999); the maximum number of incoming and outgoing citations for a single source; the fraction of all sources with at least one incoming and outgoing citation; and the percentage of original sources that had at least one incoming citation. Hub and authority scores are values between 0 and 1, computed so that the vector norm of each set of scores is equal to 1. A high hub score value for a particular source indicates that the given paper provides links to many strong authorities on the topic. A high authority score for a particular source indicates that the given paper is cited by many other sources.

While these metrics capture some important features of the citation networks, they miss others. For example, Kleinberg's authority score credits sources as being authorities only if they are cited many times but does not give credit to sources on which the putative "authorities" rely. For example, Moser (1979) is the most relied upon source of data for the incubation period of influenza counting all downstream citations. However, it is assigned a lower authority score than Cox (1999) which is cited many times but relies solely on the Moser data.

The following table displays metrics for the citation networks, by disease. The "N" column shows the total number of papers for a disease that either contained original data or had at least one incoming or outgoing citation. The "outgoing citations" columns present metrics used to characterize a network by the number of citations each paper provides for its statement of the incubation period. The "incoming citations" column present metrics used to characterize a network by the number of times each paper is cited. The maximum columns show the maximum number of incoming or outgoing citations contained in a single paper on the given disease. The "% with > 0" columns show the percent of papers that provided at least one citation for their incubation period statement or had at least one incoming citation. The maximum hub and authority score columns show the largest calculated value for an individual source, based on the algorithms developed by Kleinberg (1999).

		outgoing citations			incoming citations		
**disease**	**N**	**maximum**	**% with >0**	**maximum hub score**	**maximum**	**% with >0**	**maximum authority score**
human coronavirus	6	1	66.7	0.50	4	16.7	1.00
SARS	122	15	66.4	0.45	30	39.3	0.71
adenovirus	6	2	33.3	1.00	1	50.0	0.71
human metapneumovirus	6	1	50.0	0.58	1	50.0	0.58
influenza	77	4	48.1	0.29	14	33.8	0.99
measles	44	7	43.2	0.95	3	45.5	0.46
parainfluenza	13	4	46.2	0.85	3	69.2	0.72
respiratory syncitial virus	27	5	59.3	0.57	6	55.6	0.61
rhinovirus	18	3	27.8	0.61	3	38.9	0.73

References

JM Kleinberg. (1999) Authoritative sources in a hyperlinked environment. *JACM*. 46(5): 604–632.
