# Supplementary material for: Visualizing Clinical Evidence: Citation Networks for the Incubation Periods of Respiratory Viral Infections
Source: PLoS One. 2011 Apr 29;6(4):e19496. doi: 10.1371/journal.pone.0019496 (PMC3084881; doi:10.1371/journal.pone.0019496)
Supplement: References S1 — A complete bibliography of the sources found by the literature review. (PDF) [file pone.0019496.s004.pdf]

References used in incubation period citation networks

Supplementary information for the PLoS ONE manuscript
"Visualizing clinical evidence: citation networks for the incubation periods of respiratory viral infections"

Nicholas G. Reich, Justin Lessler, Trish M. Perl, Derek A. T. Cummings

February 4, 2011

Canonical References

[REF2] Heymann, D., et al. *Control of Communicable Diseases Manual.* American Public Health Association, 2004.

[REF3] Howley, P., Knipe, D., and Fields, B. *Fields Virology.* Lippincott Williams & Wilkins, 2007.

[REF4] Mandell, G. L., Dolin, R., and Bennett, J. E. *Principles and Practice of Infectious Disease.* Elsevier, 2005.

[REF1] Pickering, L. *Red Book: 2006 Report of the Committee on Infectious Diseases.* American Academy of Pediatrics, 2006.

Adenovirus References

[ADV63] Baum, S. *Infectious Diseases.* Saunders, 1992, ch. Adenoviruses, pp. 1663–1667.

[ADV65] BELL, J. A., ROWE, W. P., and ROSEN, L. Acute respiratory diseases of viral etiology. ii. adenoviruses. *Am J Public Health Nations Health 52* (Jun 1962), 902–907.

[ADV60] Commission on Acute Respiratory Disease. Experimental transmission of minor respiratory illness to human volunteers by filter-passing agents. i. demonstration of two types of illness characterized by long and short incubation periods and different clinical features. *J Clin Invest 26*, 5 (Sep 1947), 957–982.

[ADV1] Feikin, D. R., Moroney, J. F., Talkington, D. F., Thacker, W. L., Code, J. E., Schwartz, L. A., Erdman, D. D., Butler, J. C., and Cetron, M. S. An outbreak of acute respiratory disease caused by mycoplasma pneumoniae and adenovirus at a federal service training academy: new implications from an old scenario. *Clin Infect Dis 29*, 6 (Dec 1999), 1545–1550.

[ADV56] Foy, H. M. *Viral Infections of Humans: Epidemiology and Control.* Plenum Medical, 1997, ch. Adenoviruses, pp. 119–138.

[ADV57] Gaydos, C. A., and Gaydos, J. C. *Vaccines.* W.B. Saunders Co., 1999, ch. Adenovirus Vaccines.

[ADV53] Hayden, F. G., and Ison, M. G. Respiratory viral infections: Infections caused by specific agents. online, 6 2006. accessed 1/31/2008.

[ADV2] Huang, D. *Mucosal Immunology and Virology.* Springer London, 2006, ch. Common Respiratory Viruses and Pulmonary Mucosal Immunology, pp. 157–177.

[ADV50] Langley, J. M. Adenoviruses. *Pediatr Rev 26*, 7 (Jul 2005), 244–249.

[ADV48] McNeill, K. M., Benton, F. R., Monteith, S. C., Tuchscherer, M. A., and Gaydos, J. C. Epidemic spread of adenovirus type 4-associated acute respiratory disease between u.s. army installations. *Emerg Infect Dis 6*, 4 (2000), 415–419.

[ADV26] Ruuskanen, O., Mertsola, J., and Meurman, O. Adenovirus infection in families. *Arch Dis Child 63*, 10 (Oct 1988), 1250–1253.

[ADV36] Valenti, W. M., Menegus, M. A., Hall, C. B., Pincus, P. H., and Douglas, R. G. Nosocomial viral infections: I. epidemiology and significance. *Infect Control 1*, 1 (1980), 33–37.

Coronavirus References

[CV118] Bulliten board. *Journal of Women's Health 12* (2003), 608.

[CV165] Clinical descriptions of severe acute respiratory syndrome (sars). *Eurosurveillance Weekly 3* (2003).

[CV145] From the centers for disease control and prevention. severe acute respiratory syndrome–taiwan, 2003. *JAMA 289*, 22 (Jun 2003), 2930–2932.

[CV180] Severe acute respiratory syndrome. *Md Med 4*, 2 (2003), 50–51.

[CV248] Abdullah, A. S. M., Tomlinson, B., Cockram, C. S., and Thomas, G. N. Lessons from the severe acute respiratory syndrome outbreak in hong kong. *Emerg Infect Dis 9*, 9 (Sep 2003), 1042–1045.

[CV105] Adis International Limited. Management of sars relies on supportive measures and preventing the spread of infection. *Drugs & Therapy Perspectives 22* (2006), 16–19.

[CV81] Allen, U., Hbert, D., Churchill, C., Read, S., Bitnun, A., Richardson, S., Matlow, A., Tellier, R., Goldman, C., Wray, R., King, S., Opavsky, M. A., Ford-Jones, E., Hernandez, M., Kitai, I., Investigative, S. A. R. S., and to Transplantation, M. T. R. The potential impact of sars on organ transplantation: exercise caution. *Pediatr Transplant 7*, 5 (Oct 2003), 345–347.

[CV104] Ammon, A. *Coronaviruses with Special Emphasis on First Insights Concerning SARS*. Birkhuser Basel, 2005, ch. Disease management strategies in SARS, pp. 213–226.

[CV363] Avendano, M., Derkach, P., and Swan, S. Clinical course and management of sars in health care workers in toronto: a case series. *CMAJ 168*, 13 (Jun 2003), 1649–1660.

[CV189] Bahri, S. Enhancing quality of data through automated sars contact tracing method using rfid technology. *International Journal of Networking and Virtual Organisations 4* (2007), 145–162.

[CV199] Bakewell, S., and Blanchard, J. Information on critical issues related to infection control presented during the eighth conference on infectious diseases monday, dec 8, to wednesday, dec 10, 2003. *AORN 80* (2004), 303–310.

[CV87] Baltimore, R. S. Sars information continually updated on web. *AAP News 22* (2003).

[CV46] Berger, A., Drosten, C., Doerr, H. W., Strmer, M., and Preiser, W. Severe acute respiratory syndrome (sars)–paradigm of an emerging viral infection. *J Clin Virol 29*, 1 (Jan 2004), 13–22.

[CV93] Bever, P. V., Hia, C. P. P., and Quek, S. C. Childhood sars in singapore. *Arch Dis Child 88*, 8 (Aug 2003), 742.

[CV109] Bi, P., Wang, J., and Hiller, J. E. Weather: driving force behind the transmission of severe acute respiratory syndrome in china? *Intern Med J 37*, 8 (Aug 2007), 550–554.

[CV37] Booth, C. M., Matukas, L. M., Tomlinson, G. A., Rachlis, A. R., Rose, D. B., Dwosh, H. A., Walmsley, S. L., Mazzulli, T., Avendano, M., Derkach, P., Ephtimios, I. E., Kitai, I., Mederski, B. D., Shadowitz, S. B., Gold, W. L., Hawryluck, L. A., Rea, E., Chenkin, J. S., Cescon, D. W., Poutanen, S. M., and Detsky, A. S. Clinical features and short-term outcomes of 144 patients with sars in the greater toronto area. *JAMA 289*, 21 (Jun 2003), 2801–2809.

[CV31] Bradburne, A. F., Bynoe, M. L., and Tyrrell, D. A. Effects of a "new" human respiratory virus in volunteers. *Br Med J 3*, 5568 (Sep 1967), 767–769.

[CV197] Breimer, L., and Von Roemeling, R. Treatment of inflammatory respiratory diseases. Patent, 6 2007. United States Patent 20070141053.

[CV179] Brillman, J. C. Sars and avian flu: Respiratory diseases emerging from the far east [id rounds]. *Emergency Medicine News 26* (2004), 26–28.

[CV55] Cai, Q.-C., Xu, Q.-F., Xu, J.-M., Guo, Q., Cheng, X., Zhao, G.-M., Sun, Q.-W., Lu, J., and Jiang, Q.-W. Refined estimate of the incubation period of severe acute respiratory syndrome and related influencing factors. *Am J Epidemiol 163*, 3 (Feb 2006), 211–216.

[CV204] Caribean Epidemiology Centere. Severe acute respiratory syndrome (sars) outbreak - global. *CAREC Surveillance Report 23* (2003), 6–11.

[CV125] CDC. Efficiency of quarantine during an epidemic of severe acute respiratory syndrome beijing, china, 2003 (editorial note). *MMWR 52* (2003), 1037–1040.

[CV40] CDC. Updated interim surveillance case definition for severe acute respiratory syndrome (sars) — united states, april 29, 2003. *MMWR 52* (2003), 391–393.

[CV181] Centers for Disease Control and Prevention. *Public Health Guidance for Community-Level Preparedness and Response to Severe Acute Respiratory Syndrome (SARS) Version 2*. Centers for Disease Control and Prevention, 2005, ch. Appendix 1Clinical, Epidemiologic, and Virologic Features of SARS-CoV. accessed online 2/4/2008.

[CV66] Chan-Yeung, M. Severe acute respiratory syndrome (sars) and healthcare workers. *Int J Occup Environ Health 10*, 4 (2004), 421–427.

[CV16] Chan-Yeung, M., Ooi, G. C., Hui, D. S., Ho, P. L., and Tsang, K. W. Severe acute respiratory syndrome. *Int J Tuberc Lung Dis 7*, 12 (Dec 2003), 1117–1130.

[CV15] Chan-Yeung, M., and Xu, R.-H. Sars: epidemiology. *Respirology 8 Suppl* (Nov 2003), S9–14.

[CV159] Chen, K.-T., Twu, S.-J., Chang, H.-L., Wu, Y.-C., Chen, C.-T., Lin, T.-H., Olsen, S. J., Dowell, S. F., Su, I.-J., and Team, T. S. R. Sars in taiwan: an overview and lessons learned. *Int J Infect Dis 9*, 2 (Mar 2005), 77–85.

[CV224] Chen, M. I. C., Tan, I. B. H., and Ng, Y.-Y. Modelling the utility of body temperature readings from primary care consults for sars surveillance in an army medical centre. *Ann Acad Med Singapore 35*, 4 (Apr 2006), 236–241.

[CV61] Chen, Z., Zhang, L., Qin, C., Ba, L., Yi, C. E., Zhang, F., Wei, Q., He, T., Yu, W., Yu, J., Gao, H., Tu, X., Gettie, A., Farzan, M., Yuen, K.-Y., and Ho, D. D. Recombinant modified vaccinia virus ankara expressing the spike glycoprotein of severe acute respiratory syndrome coronavirus induces protective neutralizing antibodies primarily targeting the receptor binding region. *J Virol 79*, 5 (Mar 2005), 2678–2688.

[CV129] Cheng, V. C. C., Lau, S. K. P., Woo, P. C. Y., and Yuen, K. Y. Severe acute respiratory syndrome coronavirus as an agent of emerging and reemerging infection. *Clin Microbiol Rev 20*, 4 (Oct 2007), 660–694.

[CV88] Cherry, J. D., and Krogstad, P. Sars: the first pandemic of the 21st century. *Pediatr Res 56*, 1 (Jul 2004), 1–5.

[CV137] Chinese Medical Association. Consensus for the management of severe acute respiratory syndrome. *Chinese Medical Journal 116* (2003), 1693–1635.

[CV247] Choi, B. C. K., and Pak, A. W. P. A simple approximate mathematical model to predict the number of severe acute respiratory syndrome cases and deaths. *J Epidemiol Community Health 57*, 10 (Oct 2003), 831–835.

[CV243] Chow, K. Y., Lee, C. E., Ling, M. L., Heng, D. M. K., and Yap, S. G. Outbreak of severe acute respiratory syndrome in a tertiary hospital in singapore, linked to an index patient with atypical presentation: epidemiological study. *BMJ 328*, 7433 (Jan 2004), 195.

[CV185] Chu, C., and Tsang, K. Newly emerged respiratory infections sars and h5n1. *The Journal of the Royal College of Physicians of Edinburgh 36* (2006), 245–248.

[CV364] Conly, J. M., and Johnston, B. L. Sars : A tale of two epidemics. *Can J Infect Dis 14*, 3 (May 2003), 147–149.

[CV115] Connell, J., and OReilly, C. Sars: The emergence and identification of a new human pathogen. *Virus Alert: Bulletin of the National Virus Reference Laboratory* (2003), 1–2.

[CV102] Cowling, B. J., Ho, L. M., and Leung, G. M. Effectiveness of control measures during the sars epidemic in beijing: a comparison of the rt curve and the epidemic curve. *Epidemiology and Infection* (2007).

[CV73] Cowling, B. J., Muller, M. P., Wong, I. O. L., Ho, L.-M., Louie, M., McGeer, A., and Leung, G. M. Alternative methods of estimating an incubation distribution: examples from severe acute respiratory syndrome. *Epidemiology 18*, 2 (Mar 2007), 253–259.

[CV200] De Luca, G. Interferon beta in severe acute respiratory syndrome (sars), 2 2007. United States Patent 20070026014.

[CV80] Denison, M. R. Severe acute respiratory syndrome coronavirus pathogenesis, disease and vaccines: an update. *Pediatr Infect Dis J 23*, 11 Suppl (Nov 2004), S207–S214.

[CV50] DiGiovanni, C., Conley, J., Chiu, D., and Zaborski, J. Factors influencing compliance with quarantine in toronto during the 2003 sars outbreak. *Biosecur Bioterror 2*, 4 (2004), 265–272.

[CV214] Dimitrov, D. S., Xiao, X., and Zhongyu, Z. Soluble fragments of the sars-cov spike glycoprotein, 10 2006. United States Patent 20060240515.

[CV54] Donnelly, C. A., Fisher, M. C., Fraser, C., Ghani, A. C., Riley, S., Ferguson, N. M., and Anderson, R. M. Epidemiological and genetic analysis of severe acute respiratory syndrome. *Lancet Infect Dis 4*, 11 (Nov 2004), 672–683.

[CV39] Donnelly, C. A., Ghani, A. C., Leung, G. M., Hedley, A. J., Fraser, C., Riley, S., Abu-Raddad, L. J., Ho, L.-M., Thach, T.-Q., Chau, P., Chan, K.-P., Lam, T.-H., Tse, L.-Y., Tsang, T., Liu, S.-H., Kong, J. H. B., Lau, E. M. C., Ferguson, N. M., and Anderson, R. M. Epidemiological determinants of spread of causal agent of severe acute respiratory syndrome in hong kong. *Lancet 361*, 9371 (May 2003), 1761–1766.

[CV291] Drazen, J. M. Case clusters of the severe acute respiratory syndrome. *N Engl J Med 348*, 20 (May 2003), e6–e7.

[CV266] Dwosh, H. A., Hong, H. H. L., Austgarden, D., Herman, S., and Schabas, R. Identification and containment of an outbreak of sars in a community hospital. *CMAJ 168*, 11 (May 2003), 1415–1420.

[CV150] Eysenbach, G. Sars and population health technology. *J Med Internet Res 5*, 2 (2003), e14.

[CV289] Farewell, V. T., Herzberg, A. M., James, K. W., Ho, L. M., and Leung, G. M. Sars incubation and quarantine times: when is an exposed individual known to be disease free? *Stat Med 24*, 22 (Nov 2005), 3431–3445.

[CV116] Feng, Y., and Gao, G. F. Towards our understanding of sars-cov, an emerging and devastating but quickly conquered virus. *Comparative Immunology, Microbiology and Infectious Diseases 30* (2007), 309–327.

[CV94] File, T. M., and Tsang, K. W. T. Severe acute respiratory syndrome: pertinent clinical characteristics and therapy. *Treat Respir Med 4*, 2 (2005), 95–106.

[CV152] Foo, C.-L., Tham, K.-Y., and Seow, E. Evolution of an emergency department screening questionnaire for severe acute respiratory syndrome. *Acad Emerg Med 11*, 2 (Feb 2004), 156–161.

[CV172] for Disease Control, C., and (CDC), P. Preliminary clinical description of severe acute respiratory syndrome. *MMWR Morb Mortal Wkly Rep 52*, 12 (Mar 2003), 255–256.

[CV286] for Disease Control, C., and (CDC), P. Severe acute respiratory syndrome–singapore, 2003. *MMWR Morb Mortal Wkly Rep 52*, 18 (May 2003), 405–411.

[CV164] Freedman, D. O. Sars-lessons learned so far. *Travel Med Infect Dis 1*, 2 (May 2003), 67–68.

[CV146] Galvani, A. P., Lei, X., and Jewell, N. P. Severe acute respiratory syndrome: temporal stability and geographic variation in case-fatality rates and doubling times. *Emerg Infect Dis 9*, 8 (Aug 2003), 991–994.

[CV68] Groneberg, D. A., Hilgenfeld, R., and Zabel, P. Molecular mechanisms of severe acute respiratory syndrome (sars). *Respir Res 6* (2005), 8.

[CV67] Groneberg, D. A., Zhang, L., Welte, T., Zabel, P., and Chung, K. F. Severe acute respiratory syndrome: global initiatives for disease diagnosis. *QJM 96*, 11 (Nov 2003), 845–852.

[CV108] Hayden, F. G., and Ison, M. G. Respiratory viral infections: Infections caused by specific agents. *ACP Medicine Online* (2006). Accessed online January 2008.

[CV4] Hayden, F. G., and Ison, M. G. Respiratory viral infections: Infections caused by specific agents. *ACP Medicine Online* (2006). Accessed online January 2008.

[CV170] Henley, E. Sars: Lessons learned thus far. *The Journal of Family Practice 52* (2003), 528–530.

[CV208] Herron Jr., R. H. Door handle cover, 1 2006. United States Patent 20060006678.

[CV114] Hoffmann, C., and Kamps, B. S. *SARSReference.com*. Flying Publisher, 2003, ch. Clinical Presentation and Diagnosis, pp. 124–143.

[CV160] Houng, H.-S. H., Norwood, D., Ludwig, G. V., Sun, W., Lin, M., and Vaughn, D. W. Development and evaluation of an efficient 3'-noncoding region based sars coronavirus (sars-cov) rt-pcr assay for detection of sars-cov infections. *J Virol Methods 120*, 1 (Sep 2004), 33–40.

[CV113] Hsu, C.-Y., Chang, L.-P., and Wang, T.-L. Emerging infectious disease (3): Severe acute respiratory syndrome. *Annals of Disaster Medicine 3 Suppl 2* (2005), S52–S66.

[CV299] Hsu, L.-Y., Lee, C.-C., Green, J. A., Ang, B., Paton, N. I., Lee, L., Villacian, J. S., Lim, P.-L., Earnest, A., and Leo, Y.-S. Severe acute respiratory syndrome (sars) in singapore: clinical features of index patient and initial contacts. *Emerg Infect Dis 9*, 6 (Jun 2003), 713–717.

[CV124] Huang, D. *Mucosal Immunology and Virology.* Springer London, 2006, ch. Common Respiratory Viruses and Pulmonary Mucosal Immunology, pp. 157–177.

[CV191] Hughes, J. M. Sars: An emerging global microbial threat. *Trans Am Clin Climatol Assoc 115* (2004), 361–374.

[CV70] Hui, D. S. C., Chan, M. C. H., Wu, A. K., and Ng, P. C. Severe acute respiratory syndrome (sars): epidemiology and clinical features. *Postgrad Med J 80*, 945 (Jul 2004), 373–381.

[CV52] Hui, D. S. C., and Sung, J. J. Y. Severe acute respiratory syndrome. *Chest 124*, 1 (Jul 2003), 12–15.

[CV53] Hui, D. S.-C., Wong, P.-C., and Wang, C. Sars: clinical features and diagnosis. *Respirology 8 Suppl* (Nov 2003), S20–S24.

[CV149] Isakbaeva, E. T., Khetsuriani, N., Beard, R. S., Peck, A., Erdman, D., Monroe, S. S., Tong, S., Ksiazek, T. G., Lowther, S., Pandya-Smith, I., Anderson, L. J., Lingappa, J., Widdowson, M.-A., and Group, S. A. R. S. I. Sars-associated coronavirus transmission, united states. *Emerg Infect Dis 10*, 2 (Feb 2004), 225–231.

[CV207] Jacobs, L. A. Rights and quarantine during the sars global health crisis: Differentiated legal consciousness in hong kong, shanghai, and toronto. *Law & Society Review 41* (2007), 511–552.

[CV147] Jernigan, J. A., Low, D. E., and Hefland, R. F. Combining clinical and epidemiologic features for early recognition of sars. *Emerg Infect Dis 10*, 2 (Feb 2004), 327–333.

[CV75] Juang, J.-L., Chen, T.-C., Jiang, S. S., Hsiung, C. A., Chen, W.-C., Chen, G.-W., Lin, S.-M., Lin, J.-H., Chiu, S.-C., and Lai, Y.-K. Coupling multiplex rt-pcr to a gene chip assay for sensitive and semiquantitative detection of severe acute respiratory syndrome-coronavirus. *Lab Invest 84*, 9 (Sep 2004), 1085–1091.

[CV305] Kaslow, D. C. Biological feasibility of developing prophylactic vaccines for viral pathogens: incubation period as a critical parameter. *Hum Vaccin 3*, 1 (2007), 1–7.

[CV156] Keeler, N., and Lingappa, J. Severe acute respiratory syndrome: public health response and clinical practice update for an emerging disease. *Curr Opin Pediatr 16*, 1 (Feb 2004), 61–69.

[CV106] Kissoon, N. Severe acute respiratory syndrome: Providing care while minimizing personal risks. *Indian Pediatrics 40* (2003), 645–651.

[CV95] Kong, T. K., Dai, D. L. K., Leung, M. F., Au, S. Y., Yung, R., and Chan, M. H. Severe acute respiratory syndrome (sars) in elders. *J Am Geriatr Soc 51*, 8 (Aug 2003), 1182–1183.

[CV9] Kuk, A. Y. C., and Ma, S. The estimation of sars incubation distribution from serial interval data using a convolution likelihood. *Stat Med 24*, 16 (Aug 2005), 2525–2537.

[CV57] Lai, P. C., Wong, C. M., Hedley, A. J., Lo, S. V., Leung, P. Y., Kong, J., and Leung, G. M. Understanding the spatial clustering of severe acute respiratory syndrome (sars) in hong kong. *Environ Health Perspect 112*, 15 (Nov 2004), 1550–1556.

[CV158] Lapinsky, S. E., and Granton, J. T. Critical care lessons from severe acute respiratory syndrome. *Curr Opin Crit Care 10*, 1 (Feb 2004), 53–58.

[CV36] Lee, N., Hui, D., Wu, A., Chan, P., Cameron, P., Joynt, G. M., Ahuja, A., Yung, M. Y., Leung, C. B., To, K. F., Lui, S. F., Szeto, C. C., Chung, S., and Sung, J. J. Y. A major outbreak of severe acute respiratory syndrome in hong kong. *N Engl J Med 348*, 20 (May 2003), 1986–1994.

[CV276] Lee, P. O., Tsui, P. T., Tsang, T. Y., Chau, T. N., Kwan, C. P., Yu, W. C., and Lai, S. T. *Coronaviruses with Special Emphasis on First Insights Concerning SARS*. Birkhuser Basel, 2005, ch. Severe acute respiratory syndrome: clinical features, pp. 71–85.

[CV293] Lee, P. O., Tsui, P. T., Tsang, T. Y., Chau, T. N., Kwan, C. P., Yu, W. C., and Lai, S. T. *Coronaviruses with Special Emphasis on First Insights Concerning SARS*. Birkhuser Basel, 2005, ch. Severe acute respiratory syndrome: clinical features, pp. 71–85.

[CV74] Leong, W. F., Tan, H. C., Ooi, E. E., Koh, D. R., and Chow, V. T. K. Microarray and real-time rt-pcr analyses of differential human gene expression patterns induced by severe acute respiratory syndrome (sars) coronavirus infection of vero cells. *Microbes Infect 7*, 2 (Feb 2005), 248–259.

[CV234] Leung, G. M., Hedley, A. J., Ho, L.-M., Chau, P., Wong, I. O. L., Thach, T. Q., Ghani, A. C., Donnelly, C. A., Fraser, C., Riley, S., Ferguson, N. M., Anderson, R. M., Tsang, T., Leung, P.-Y., Wong, V., Chan, J. C. K., Tsui, E., Lo, S.-V., and Lam, T.-H. The epidemiology of severe acute respiratory syndrome in the 2003 hong kong epidemic: an analysis of all 1755 patients. *Ann Intern Med 141*, 9 (Nov 2004), 662–673.

[CV62] Leung, T. F., Wong, G. W. K., Hon, K. L. E., and Fok, T. F. Severe acute respiratory syndrome (sars) in children: epidemiology, presentation and management. *Paediatr Respir Rev 4*, 4 (Dec 2003), 334–339.

[CV201] Leung, T. M. D., Tam, C. H. F., Ma, C. H., Lim, P. L., and Chan, K. S. P. Compositions and methods for diagnosing and preventing severe acute respiratory syndrome (sars), 5 2005. United States Patent 20050112559.

[CV86] Li, A. M., and Ng, P. C. Severe acute respiratory syndrome (sars) in neonates and children. *Arch Dis Child Fetal Neonatal Ed 90*, 6 (Nov 2005), F461–F465.

[CV103] Li, G. Modeling the effect of information feedback on the sars epidemic in beijing. In *The 23rd International Conference of the System Dynamics Society* (2005).

[CV42] Li, T., Qiu, Z., Zhang, L., Han, Y., He, W., Liu, Z., Ma, X., Fan, H., Lu, W., Xie, J., Wang, H., Deng, G., and Wang, A. Significant changes of peripheral t lymphocyte subsets in patients with severe acute respiratory syndrome. *J Infect Dis 189*, 4 (Feb 2004), 648–651.

[CV301] Lipsitch, M., Cohen, T., Cooper, B., Robins, J. M., Ma, S., James, L., Gopalakrishna, G., Chew, S. K., Tan, C. C., Samore, M. H., Fisman, D., and Murray, M. Transmission dynamics and control of severe acute respiratory syndrome. *Science 300*, 5627 (Jun 2003), 1966–1970.

[CV209] Lloyd-Smith, J. O. *Disease transmission in heterogeneous populations*. PhD thesis, University of California, Berkeley, 2005.

[CV210] Lodhi, Z.-E.-H. Severe acute respiratory syndrome (sars). *The Voice Magazine 11* (2003), 18–20.

[CV91] Lopez, V., Chan, K. S., and Wong, Y. C. J. Nursing care of patients with severe acute respiratory syndrome in the intensive care unit: case reports in hong kong. *Int J Nurs Stud 41*, 3 (Mar 2004), 263–272.

[CV141] Low, D. E., and McGeer, A. Sars–one year later. *N Engl J Med 349*, 25 (Dec 2003), 2381–2382.

[CV60] Maki, D. G. Sars: 1918 revisited? the urgent need for global collaboration in public health. *Mayo Clin Proc 78*, 7 (Jul 2003), 813–816.

[CV84] Maki, D. G. Sars revisited: the challenge of controlling emerging infectious diseases at the local, regional, federal, and global levels. *Mayo Clin Proc 79*, 11 (Nov 2004), 1359–1366.

[CV154] Manocha, S., Walley, K. R., and Russell, J. A. Severe acute respiratory distress syndrome (sars): a critical care perspective. *Crit Care Med 31*, 11 (Nov 2003), 2684–2692.

[CV112] Marley, C. T., Levsky, M. E., Talbot, T. S., and Kang, C. S. Sars and its impact on current and future emergency department operations. *J Emerg Med 26*, 4 (May 2004), 415–420.

[CV202] Mazzulli, T., Kain, K., and Butany, J. Severe acute respiratory syndrome: overview with an emphasis on the toronto experience. *Arch Pathol Lab Med 128*, 12 (Dec 2004), 1346–1350.

[CV76] McBryde, E. S., Gibson, G., Pettitt, A. N., Zhang, Y., Zhao, B., and McElwain, D. L. S. Bayesian modelling of an epidemic of severe acute respiratory syndrome. *Bull Math Biol 68*, 4 (May 2006), 889–917.

[CV85] Meissner, H. C. Reducing the impact of viral respiratory infections in children. *Pediatr Clin North Am 52*, 3 (Jun 2005), 695–710, v.

[CV44] Meltzer, M. I. Multiple contact dates and sars incubation periods. *Emerg Infect Dis 10*, 2 (Feb 2004), 207–209.

[CV203] Miller, R. I. The impact of quarantine on military operations. Tech. rep., USAF Counterproliferation Center, 2005.

[CV48] ming Chan, W., wah Kwan, Y., shan Wan, H., wai Leung, C., and chun Chiu, M. Epidemiologic linkage and public health implication of a cluster of severe acute respiratory syndrome in an extended family. *Pediatr Infect Dis J 23*, 12 (Dec 2004), 1156–1159.

[CV6] Ng, S. K. C. Possible role of an animal vector in the sars outbreak at amoy gardens. *Lancet 362*, 9383 (Aug 2003), 570–572.

[CV144] Ng, T. W., Turinici, G., and Danchin, A. A double epidemic model for the sars propagation. *BMC Infect Dis 3* (Sep 2003), 19.

[CV143] Olsen, S. J., Chang, H.-L., Cheung, T. Y.-Y., Tang, A. F.-Y., Fisk, T. L., Ooi, S. P.-L., Kuo, H.-W., Jiang, D. D.-S., Chen, K.-T., Lando, J., Hsu, K.-H., Chen, T.-J., and Dowell, S. F. Transmission of the severe acute respiratory syndrome on aircraft. *N Engl J Med 349*, 25 (Dec 2003), 2416–2422.

[CV10] Oxford, J. S., Balasingam, S., Chan, C., Catchpole, A., and Lambkin, R. New antiviral drugs, vaccines and classic public health interventions against sars coronavirus. *Antivir Chem Chemother 16*, 1 (2005), 13–21.

[CV290] Peiris, J. S. M., Yuen, K. Y., Osterhaus, A. D. M. E., and Sthr, K. The severe acute respiratory syndrome. *N Engl J Med 349*, 25 (Dec 2003), 2431–2441.

[CV166] Peleman, R. A. New and re-emerging infectious diseases: epidemics in waiting. *Curr Opin Anaesthesiol 17*, 3 (Jun 2004), 265–270.

[CV58] Poutanen, S., and McGeer, A. Transmission and control of sars. *Curr Infect Dis Rep 6*, 3 (Jun 2004), 220–227.

[CV295] Poutanen, S. M., Low, D. E., Henry, B., Finkelstein, S., Rose, D., Green, K., Tellier, R., Draker, R., Adachi, D., Ayers, M., Chan, A. K., Skowronski, D. M., Salit, I., Simor, A. E., Slutsky, A. S., Doyle, P. W., Krajden, M., Petric, M., Brunham, R. C., McGeer, A. J., National Microbiology Laboratory, C., and Team, C. S. A. R. S. S. Identification of severe acute respiratory syndrome in canada. *N Engl J Med 348*, 20 (May 2003), 1995–2005.

[CV292] Public Health Agency of Canada. Summary of severe acute respiratory syndrome (sars) cases: Canada and international (april 2, 2003). web, April 2003. accessed 2/4/2008.

[CV366] Public Health Agency of Canada. Summary of severe acute respiratory syndrome (sars) cases: Canada and international (april 29, 2003). web, April 2003. accessed 2/4/2008.

[CV287] Riley, S., Fraser, C., Donnelly, C. A., Ghani, A. C., Abu-Raddad, L. J., Hedley, A. J., Leung, G. M., Ho, L.-M., Lam, T.-H., Thach, T. Q., Chau, P., Chan, K.-P., Lo, S.-V., Leung, P.-Y., Tsang, T., Ho, W., Lee, K.-H., Lau, E. M. C., Ferguson, N. M., and Anderson, R. M. Transmission dynamics of the etiological agent of sars in hong kong: impact of public health interventions. *Science 300*, 5627 (Jun 2003), 1961–1966.

[CV157] Roberts, A., Thomas, W. D., Guarner, J., Lamirande, E. W., Babcock, G. J., Greenough, T. C., Vogel, L., Hayes, N., Sullivan, J. L., Zaki, S., Subbarao, K., and Ambrosino, D. M. Therapy with a severe acute respiratory syndrome-associated coronavirus-neutralizing human monoclonal antibody reduces disease severity and viral burden in golden syrian hamsters. *J Infect Dis 193*, 5 (Mar 2006), 685–692.

[CV110] Ryan, C. P. Where do pets fit into human quarantines? *J Public Health (Oxf) 29*, 1 (Mar 2007), 70–71.

[CV198] Samaranayake, L. P., and Peiris, M. Severe acute respiratory syndrome and dentistry: a retrospective view. *J Am Dent Assoc 135*, 9 (Sep 2004), 1292–1302.

[CV294] Scales, D. C., Green, K., Chan, A. K., Poutanen, S. M., Foster, D., Nowak, K., Raboud, J. M., Saskin, R., Lapinsky, S. E., and Stewart, T. E. Illness in intensive care staff after brief exposure to severe acute respiratory syndrome. *Emerg Infect Dis 9*, 10 (Oct 2003), 1205–1210.

[CV117] Scrimini, S., Junemann, A., and Luna, C. M. Community acquired pneumonia in the tropics. *Curr Opin Pulm Med 13*, 3 (May 2007), 170–176.

[CV51] Skowronski, D. M., Astell, C., Brunham, R. C., Low, D. E., Petric, M., Roper, R. L., Talbot, P. J., Tam, T., and Babiuk, L. Severe acute respiratory syndrome (sars): a year in review. *Annu Rev Med 56* (2005), 357–381.

[CV11] So, R. C. H., Ko, J., Yuan, Y. W. Y., Lam, J. J., and Louie, L. Severe acute respiratory syndrome and sport: facts and fallacies. *Sports Med 34*, 15 (2004), 1023–1033.

[CV163] Stadler, K., and Rappuoli, R. Sars: understanding the virus and development of rational therapy. *Curr Mol Med 5*, 7 (Nov 2005), 677–697.

[CV100] Stockman, L. J., and Parashar, U. D. Review of the epidemiology of severe acute respiratory syndrome (sars). *BUSINESS BRIEFING: CLINICAL VIROLOGY & INFECTIOUS DISEASE* (2004).

[CV288] Svoboda, T., Henry, B., Shulman, L., Kennedy, E., Rea, E., Ng, W., Wallington, T., Yaffe, B., Gournis, E., Vicencio, E., Basrur, S., and Glazier, R. H. Public health measures to control the spread of the severe acute respiratory syndrome during the outbreak in toronto. *N Engl J Med 350*, 23 (Jun 2004), 2352–2361.

[CV120] Syed, S., and Saleem, A. Severe acute respiratory syndrome epidemiology and control. *Laboratory Medicine* (2004), 112–116.

[CV216] Sderbaum, F., and Hettne, B. Regional cooperation and the provision of regional and global public goods, 5 2005. For the seminar on Competition and complementarity between global and regional public goods.

[CV174] Talekar, S. Report on severe acute respiratory distress syndrome (”sars”) - are we really dealing with a new syndrome? *Indian Journal of Critical Care Medicine 7* (2003), 10–13.

[CV218] Tam, W. W., and Wong, T. W. Estimating incubation period with multiple contact days. *Scand J Infect Dis 39*, 6-7 (2007), 609–611.

[CV63] Tan, J., Mu, L., Huang, J., Yu, S., Chen, B., and Yin, J. An initial investigation of the association between the sars outbreak and weather: with the view of the environmental temperature and its variation. *J Epidemiol Community Health 59*, 3 (Mar 2005), 186–192.

[CV272] Tan, T. T., Tan, B. H., Kurup, A., Oon, L. L. E., Heng, D., Thoe, S. Y. S., Bai, X. L., Chan, K. P., and Ling, A. E. Atypical sars and escherichia coli bacteremia. *Emerg Infect Dis 10*, 2 (Feb 2004), 349–352.

[CV190] Tang, H. L., Cheuk, A., Chu, K. H., Lee, W., Wong, S. H., Cheng, Y. L., Yu, A. W. Y., Fung, K. S., Tsang, W. K., Chan, H. W. H., and Tong, K. L. Severe acute respiratory syndrome in haemodialysis patients: a report of two cases. *Nephrol Dial Transplant 18*, 10 (Oct 2003), 2178–2181.

[CV128] TECHNICAL WORKING GROUP ON SARS CLINICAL GUIDELINES. Interim clinical guidelines on severe acute respiratory syndrome (sars) for health facilities in the philippines - abridged version. Tech. rep., Philippines Department of Health, 2003.

[CV77] Tice, A. D., Kishimoto, M., Dinh, C. H., Lam, G. T.-K., and Marineau, M. Knowledge of severe acute respiratory syndrome among community physicians, nurses, and emergency medical responders. *Prehosp Disaster Med 21*, 3 (2006), 183–189.

[CV267] Tsang, K. W., Ho, P. L., Ooi, G. C., Yee, W. K., Wang, T., Chan-Yeung, M., Lam, W. K., Seto, W. H., Yam, L. Y., Cheung, T. M., Wong, P. C., Lam, B., Ip, M. S., Chan, J., Yuen, K. Y., and Lai, K. N. A cluster of cases of severe acute respiratory syndrome in hong kong. *N Engl J Med 348*, 20 (May 2003), 1977–1985.

[CV21] Tyrrell, D. A., Cohen, S., and Schlarb, J. E. Signs and symptoms in common colds. *Epidemiol Infect 111*, 1 (Aug 1993), 143–156.

[CV211] Valentino, L. A., and Oza, V. M. Blood safety and the choice of anti-hemophilic factor concentrate. *Pediatr Blood Cancer 47*, 3 (Sep 2006), 245–254.

[CV251] Varia, M., Wilson, S., Sarwal, S., McGeer, A., Gournis, E., Galanis, E., Henry, B., and Team, H. O. I. Investigation of a nosocomial outbreak of severe acute respiratory syndrome (sars) in toronto, canada. *CMAJ 169*, 4 (Aug 2003), 285–292.

[CV79] Venkatesh, S., and Memish, Z. A. Sars: the new challenge to international health and travel medicine. *East Mediterr Health J 10*, 4-5 (2004), 655–662.

[CV7] wai Wong, T., kei Lee, C., Tam, W., fai Lau, J. T., sun Yu, T., fai Lui, S., Chan, P. K. S., Li, Y., Bresee, J. S., Sung, J. J. Y., Parashar, U. D., and Group, O. S. Cluster of sars among medical students exposed to single patient, hong kong. *Emerg Infect Dis 10*, 2 (Feb 2004), 269–276.

[CV13] wai Wong, T., and Tam, W. Estimating sars incubation period. *Emerg Infect Dis 10*, 8 (Aug 2004), 1503–4; author reply 1504.

[CV49] Wang, J., Wen, J., Li, J., Yin, J., Zhu, Q., Wang, H., Yang, Y., Qin, E., You, B., Li, W., Li, X., Huang, S., Yang, R., Zhang, X., Yang, L., Zhang, T., Yin, Y., Cui, X., Tang, X., Wang, L., He, B., Ma, L., Lei, T., Zeng, C., Fang, J., Yu, J., Wang, J., Yang, H., West, M. B., Bhatnagar, A., Lu, Y., Xu, N., and Liu, S. Assessment of immunoreactive synthetic peptides from the structural proteins of severe acute respiratory syndrome coronavirus. *Clin Chem 49*, 12 (Dec 2003), 1989–1996.

[CV56] Wang, J.-T., and Chang, S.-C. Severe acute respiratory syndrome. *Curr Opin Infect Dis 17*, 2 (Apr 2004), 143–148.

[CV33] Wang, J.-T., Sheng, W.-H., Fang, C.-T., Chen, Y.-C., Wang, J.-L., Yu, C.-J., Chang, S.-C., and Yang, P.-C. Clinical manifestations, laboratory findings, and treatment outcomes of sars patients. *Emerg Infect Dis 10*, 5 (May 2004), 818–824.

[CV71] Wong, G. W., and Fok, T. F. Severe acute respiratory syndrome (sars) in children. *Pediatr Pulmonol Suppl 26* (2004), 69–71.

[CV283] World Health Organization. Consensus document on the epidemiology of severe acute respiratory syndrome (sars). Tech. rep., World Health Organization, 2003.

[CV285] World Health Organization. Update 49 - sars case fatality ratio, incubation period. website, May 2003. Accessed 1/18/2008.

[CV72] World Health Orginization. Preliminary clinical description of severe acute respiratory syndrome. Online, 3 2003.

[CV365] World Health Orginization. Update 27 - one month into the global sars outbreak: Status of the outbreak and lessons for the immediate future. Online, April 2003. Accessed online January 2008.

[CV41] World Health Orginization. Update 27 - one month into the global sars outbreak: Status of the outbreak and lessons for the immediate future. Online, April 2003. Accessed online January 2008.

[CV258] Wu, W., Wang, J., Liu, P., Chen, W., Yin, S., Jiang, S., Yan, L., Zhan, J., Chen, X., Li, J., Huang, Z., and Huang, H. A hospital outbreak of severe acute respiratory syndrome in guangzhou, china. *Chin Med J (Engl) 116*, 6 (Jun 2003), 811–818.

[CV155] yan Che, X., Di, B., ping Zhao, G., di Wang, Y., wen Qiu, L., Hao, W., Wang, M., zhe Qin, P., fei Liu, Y., hong Chan, K., Cheng, V. C. C., and yung Yuen, K. A patient with asymptomatic severe acute respiratory syndrome (sars) and antigenemia from the 2003-2004 community outbreak of sars in guangzhou, china. *Clin Infect Dis 43*, 1 (Jul 2006), e1–e5.

[CV59] Zakhartchouk, A. N., Liu, Q., Petric, M., and Babiuk, L. A. Augmentation of immune responses to sars coronavirus by a combination of dna and whole killed virus vaccines. *Vaccine 23*, 35 (Aug 2005), 4385–4391.

[CV32] Zhao, Z., Zhang, F., Xu, M., Huang, K., Zhong, W., Cai, W., Yin, Z., Huang, S., Deng, Z., Wei, M., Xiong, J., and Hawkey, P. M. Description and clinical treatment of an early outbreak of severe acute respiratory syndrome (sars) in guangzhou, pr china. *J Med Microbiol 52*, Pt 8 (Aug 2003), 715–720.

[CV96] Zhou, J., Gong, J., and Li, W. Human daily behavior based simulation for epidemic transmission: A case study of sars. In *Artificial Reality and Telexistence–Workshops, 2006. ICAT '06. 16th International Conference on* (Nov. 2006), pp. 589–593.

[CV168] Zoidis, J. D., and Braun, P. C. Sars: What we've learned so far. *RT* (2003). accessed online 1/4/2008.

Human Metapneumovirus References

[hMPV3] Human metapneumovirus infections cause similar symptoms and clinical severity as respiratory syncytial virus infections. *Eur J Pediatr 165*, 7 (Jul 2006), 467–475.

[hMPV2] Ebihara, T., Endo, R., Ishiguro, N., Nakayama, T., Sawada, H., and Kikuta, H. Early reinfection with human metapneumovirus in an infant. *J Clin Microbiol 42*, 12 (Dec 2004), 5944–5946.

[hMPV6] Ebihara, T., Endo, R., Kikuta, H., Ishiguro, N., Ishiko, H., Hara, M., Takahashi, Y., and Kobayashi, K. Human metapneumovirus infection in japanese children. *J Clin Microbiol 42*, 1 (Jan 2004), 126–132.

[hMPV1] Esper, F., Boucher, D., Weibel, C., Martinello, R. A., and Kahn, J. S. Human metapneumovirus infection in the united states: clinical manifestations associated with a newly emerging respiratory infection in children. *Pediatrics 111*, 6 Pt 1 (Jun 2003), 1407–1410.

[hMPV5] Peiris, J. S. M., Tang, W.-H., Chan, K.-H., Khong, P.-L., Guan, Y., Lau, Y.-L., and Chiu, S. S. Children with respiratory disease associated with metapneumovirus in hong kong. *Emerg Infect Dis 9*, 6 (Jun 2003), 628–633.

[hMPV4] Schildgen, O., Simon, A., Wilkesmann, A., Williams, J., et al. The human metapneumovirus: biology, epidemiological features and clinical characteristic of infection. *Rev Med Microbiol 17* (2006), 11–25.

Influenza References

[FLU109] AORN Guidance Statement: Human and Avian Influenza and Severe Acute Respiratory Syndrome. *AORN Journal 84*, 2 (2006), 284–298.

[FLU114] Adams, B. Influenza and its growing importance: an investigative compilation. `http://www.indiana.edu/\%7Epirt/flu/flufull5.pdf` [Accessed 24 March 2008].

[FLU135] Ahillen, C. *Agent-based moeling of the spread of the 1918–1919 Spanish Flu in three Canadian fur trading communities.* PhD thesis, University of Missouri, 2006.

[FLU36] Alford, R. H., Kasel, J. A., Gerone, P. J., and Knight, V. Human influenza resulting from aerosol inhalation. *Proc Soc Exp Biol Med 122*, 3 (Jul 1966), 800–804.

[FLU11] AlSaleh, E., Al Mazroua, M., Choudhry, A., Turkistani, A., Al Hamdan, N., Azhar, E., and Olyan, D. Serotypes of influenza during Hajj season, 1424. *Saudi Epidemiology Bulletin 12*, 1 (2005).

[FLU48] Armstrong, C., and Hopkins, R. An epidemiologic study of the 1920 epidemic of influenza in an isolated rural community. *Public Health Reports* (1921), 1671–1702.

[FLU118] Aventis. Influenza virus vaccine (fluzone). `http://www.vaccinesafety.edu/PackageInserts/AP-Fluzone.pdf` [Accessed 24 March 2004], 2003.

[FLU74] Banning, M. Influenza: incidence, symptoms and treatment. *Br J Nurs 14*, 22 (2005), 1192–1197.

[FLU7] Barlow, G., and Nathwani, D. Nosocomial influenza infection. *Lancet 355*, 9210 (Apr 2000), 1187.

[FLU98] Blaiss, M. Influenza Prevention and Treatment: New Options. Medscape Today [Accessed 22 April 2008].

[FLU89] Block, S. L. Role of influenza vaccine for healthy children in the us. *Paediatr Drugs 6*, 4 (2004), 199–209.

[FLU21] Bolyard, E., Tablan, O., Williams, W., Pearson, M., Shapiro, C., and Deitchman, S. Guideline for infection control in health care personnel, 1998. *American Journal of Infection Control 26*, 3 (1998), 289–354.

[FLU91] Boyd, M., Clezy, K., Lindley, R., and Pearce, R. Pandemic influenza: clinical issues. *Med J Aust 185*, 10 Suppl (Nov 2006), S44–S47.

[FLU176] Bridges, C., Fukuda, K., Cox, N., and Singleton, J. Prevention and Control of Influenza. *Recommendations of the Advisory Committee on immunization practices, MMWR 50* (2001), 1–46.

[FLU137] Bridges, C., Fukuda, K., Uyeki, T., Cox, N., and Singleton, J. Prevention and Control of Influenza. *Recommendations of the Advisory Committee on immunization practices, MMWR 51* (2002), 1–31.

[FLU87] Bridges, C., Fukuda, K., Uyeki, T., Cox, N., and Singleton, J. Prevention and Control of Influenza. *Recommendations of the Advisory Committee on immunization practices, MMWR 54* (2005), 1–40.

[FLU128] Bridges, C., Harper, S., Fukuda, K., Uyeki, T., Cox, N., and Singleton, J. Prevention and Control of Influenza. *Recommendations of the Advisory Committee on immunization practices, MMWR 52* (2003), 1–36.

[FLU42] Burnet, F. M., and Foley, M. The results of intranasal inoculation of modified and unmodified influenza virus strains in human volunteers. *The Medical Journal of Australia* (1940), 655–659.

[FLU142] Cherry, J. D. *Textbook of Pediatrics*, 12th ed. WB Saunders, Co., 1983, ch. Influenza viral infections.

[FLU58] Couch, R. B., Douglas, R. G., Fedson, D. S., and Kasel, J. A. Correlated studies of a recombinant influenza-virus vaccine. 3. protection against experimental influenza in man. *J Infect Dis 124*, 5 (Nov 1971), 473–480.

[FLU55] Cox, N., and Fukuda, K. Influenza. *Infectious Disease Clinics of North America 12*, 1 (1998), 27–38.

[FLU130] Cox, N., and Kawaoka, Y. Orthomyxoviruses: influenza. *Topley and Wilson's Microbiology and Microbial Infections* (1998), 385–433.

[FLU28] Cox, N. J., and Subbarao, K. Influenza. *Lancet 354*, 9186 (Oct 1999), 1277–1282.

[FLU2] Cox, R. J., Brokstad, K. A., and Ogra, P. Influenza virus: immunity and vaccination strategies. comparison of the immune response to inactivated and live, attenuated influenza vaccines. *Scand J Immunol 59*, 1 (Jan 2004), 1–15.

[FLU216] Criswell, B. S., Couch, R. B., Greenberg, S. B., and Kimzey, S. L. The lymphocyte response to influenza in humans. *Am Rev Respir Dis 120*, 3 (Sep 1979), 700–704.

[FLU64] Cumpston, J. Influenza and maritime quarantine in australia. *Commonwealth of Australia Quarantine Service, Service Publication No.18, Melbourne, Australia, Albert J. Mullet, Government Printer* (1919).

[FLU152] Cunha, B. Influenza: historical aspects of epidemics and pandemics. *Infectious Disease Clinics of North America 18*, 1 (2004), 141–155.

[FLU103] Daly, P. Pandemic influenza and physician offices. *BC Med J 49*, 5 (2007), 263.

[FLU4] Davis, L. E., Caldwell, G. G., Lynch, R. E., Bailey, R. E., and Chin, T. D. Hong kong influenza: the epidemiologic features of a high school family study analyzed and compared with a similar study during the 1957 asian influenza epidemic. *Am J Epidemiol 92*, 4 (Oct 1970), 240–247.

[FLU175] Deutsche Gesellschaft für Technische Zusammenarbeit. Health, Population, and Nutrition News & Notes. [Online accessed 17 April 2008], March 2005.

[FLU8] Diniz, E., Vieira, R., Ishida, M., Ceccon, M., Krebs, V., Feferbaum, R., and Vaz, F. Infection by influenza A virus in preterm newborn infants. *Prenatal and Neonatal Medicine 6*, 6 (2001), 358–362.

[FLU38] Douglas, R. *The Influenza Viruses and Influenza.* Academic Press, 1975, ch. 13, pp. 395–447.

[FLU75] Drinka, P. J., and Haupt, T. Emergence of rimantadine-resistant virus within 6 days of starting rimantadine prophylaxis with oseltamivir treatment of symptomatic cases. *J Am Geriatr Soc 55*, 6 (Jun 2007), 923–926.

[FLU32] Fauci, A., Braunwald, E., Kasper, D., Hauser, S., Longo, D., Jameson, J., and Loscalzo, J., Eds. *Harrison's principles of internal medicine*, 17th ed. New York: McGraw-Hill, 2001, ch. 39: Orthomyxoviruses (Influenza Viruses).

[FLU9] Feder, H. M. Zanamivir to prevent influenza. *N Engl J Med 344*, 7 (Feb 2001), 528; author reply 529–528; author reply 530.

[FLU90] Feigin, R., and Cherry, J., Eds. *Textbook of Pediatrics*, 4th ed. WB Saunders, Co., 1998, pp. 2024–2040.

[FLU57] Ferguson, N. M., Cummings, D. A. T., Cauchemez, S., Fraser, C., Riley, S., Meeyai, A., Iamsirithaworn, S., and Burke, D. S. Strategies for containing an emerging influenza pandemic in southeast asia. *Nature 437*, 7056 (Sep 2005), 209–214.

[FLU174] Fiore, A., Shay, D., Haber, P., Iskander, J., Uyeki, T., Mootrey, G., Bresee, J., and Cox, N. Prevention and Control of Influenza. *Recommendations of the Advisory Committee on immunization practices, MMWR 56* (2007), 1–54.

[FLU151] Foster, M., and Cookson, H. Observations on a small localised epidemic of influenza. *Lancet ii* (November 1918), 588–590.

[FLU84] Foy, H. M., Cooney, M. K., Allan, I. D., and Albrecht, J. K. Influenza b in households: virus shedding without symptoms or antibody response. *Am J Epidemiol 126*, 3 (Sep 1987), 506–515.

[FLU41] Francis, T., Pearson, H. E., Salk, J. E., and Brown, P. N. Immunity in human subjects artificially infected with influenza virus, type b. *Am J Public Health Nations Health 34*, 4 (Apr 1944), 317–334.

[FLU19] Fritz, R. S., Hayden, F. G., Calfee, D. P., Cass, L. M., Peng, A. W., Alvord, W. G., Strober, W., and Straus, S. E. Nasal cytokine and chemokine responses in experimental influenza a virus infection: results of a placebo-controlled trial of intravenous zanamivir treatment. *J Infect Dis 180*, 3 (Sep 1999), 586–593.

[FLU116] Handa, R., Teo, S., and Booy, R. Influenza: current evidence and informed predictions. *Expert Rev Vaccines 3*, 4 (Aug 2004), 443–451.

[FLU12] Hansen, L. Influenza. *American Family Physician 68*, 11 (2003), 2231–2234.

[FLU95] Harper, S., Fukuda, K., Uyeki, T., Cox, N., and Bridges, C. Prevention and Control of Influenza. *Recommendations of the Advisory Committee on immunization practices, MMWR 53* (2004), 1–40.

[FLU167] Harper, S., Klimov, A., Uyeki, T., and Fukuda, K. Influenza. *Clin Lab Med 22*, 4 (Dec 2002), 863–82, vi.

[FLU136] Hart, T. *Microterrors: The Complete Guide to Bacterial, Viral and Fungal Infections That Threaten Our Health.* Firefly Books, Limited, 2004.

[FLU107] Hawker, J., Begg, N., Blair, I., Reintjes, R., and Weinberg, J. *Communicable Disease Control Handbook.* Blackwell Science, 2001.

[FLU82] Hayden, F. *Antivirals in the Elderly.* Haworth Press, 1996, ch. Influenza in the Elderly.

[FLU111] Hayden, F., and Ison, M. *Respiratory Viral Infections.* ACP Medicine Online, 2006, ch. 7:XXV.

[FLU20] Hayden, F. G., Treanor, J. J., Fritz, R. S., Lobo, M., Betts, R. F., Miller, M., Kinnersley, N., Mills, R. G., Ward, P., and Straus, S. E. Use of the oral neuraminidase inhibitor oseltamivir in experimental human influenza: randomized controlled trials for prevention and treatment. *JAMA 282*, 13 (Oct 1999), 1240–1246.

[FLU3] Heikkinen, T., and Jrvinen, A. The common cold. *Lancet 361*, 9351 (Jan 2003), 51–59.

[FLU43] Henle, W., Henle, G., and Jr., J. S. Demonstration of the efficacy of vaccination againt influenza type a by experimental infection of human beings. *The Journal of Immunology 46* (1943), 163–175.

[FLU37] Henle, W., Henle, G., Jr., J. S., and Maris, E. P. Experimental exposure of human subjects to viruses of influenza. *The Journal of Immunology 52* (1945), 145–165.

[FLU13] Hollingsworth, T. D., Ferguson, N. M., and Anderson, R. M. Will travel restrictions control the international spread of pandemic influenza? *Nat Med 12*, 5 (May 2006), 497–499.

[FLU124] Illinois Foundation for Quality Healthcare. Adult Inpatient Influenza and Pneumococcal Vaccination Self-Study Continuing Education Course. [Accessed 9/5/2007].

[FLU140] Jain, A. K. Avian human influenza : diagnosis, case management and treatment. *J Indian Med Assoc 104*, 7 (Jul 2006), 372, 374–376, 378.

[FLU156] Jefferson, T. Influenza. BMJ Clinical Evidence [Online accessed 17 April 2008].

[FLU105] Jones, M., and Mar, C. D. Safety of neuraminidase inhibitors for influenza. *Expert Opin Drug Saf 5*, 5 (Sep 2006), 603–608.

[FLU83] JORDAN, W. S., DENNY, F. W., BADGER, G. F., CURTISS, C., DINGLE, J. H., OSEASOHN, R., and STEVENS, D. A. A study of illness in a group of cleveland families. xvii. the occurrence of asian influenza. *Am J Hyg 68*, 2 (Sep 1958), 190–212.

[FLU293] Kaslow, D. C. Biological feasibility of developing prophylactic vaccines for viral pathogens: incubation period as a critical parameter. *Hum Vaccin 3*, 1 (2007), 1–7.

[FLU146] Katagiri, S., Ohizumi, A., Ohyama, S., and Homma, M. Follow-up study of type c influenza outbreak in a children's home. *Microbiol Immunol 31*, 4 (1987), 337–343.

[FLU223] Khakpour, M., Saidi, A., and Naficy, K. Proved viraemia in asian influenza (hong kong variant) during incubation period. *Br Med J 4*, 5677 (Oct 1969), 208–209.

[FLU141] Kondo, S., and Abe, K. The effects of influenza virus infection on fev1 in asthmatic children. the time-course study. *Chest 100*, 5 (Nov 1991), 1235–1238.

[FLU99] Kordsmeier, J. Influenza vaccinations: should they be mandatory for nurses? pro. *MCN Am J Matern Child Nurs 31*, 2 (2006), 76.

[FLU17] Kupu, S. Pacific Public Health Surveillance Network Influenza Guidelines. Secretariat of the Pacific Community [Online accessed 17 April 2008], 2005.

[FLU168] Laibl, V., and Sheffield, J. The management of respiratory infections during pregnancy. *Immunol Allergy Clin North Am 26*, 1 (Feb 2006), 155–72, viii.

[FLU138] Lessler, J., Brookmeyer, R., and Perl, T. M. An evaluation of classification rules based on date of symptom onset to identify health-care associated infections. *Am J Epidemiol 166*, 10 (Nov 2007), 1220–1229.

[FLU97] Lester, S. K., and Hayney, M. S. Expanding childhood influenza immunization recommendations. *J Am Pharm Assoc (2003) 47*, 1 (2007), 104–105.

[FLU56] Longini, I. M., Halloran, M. E., Nizam, A., and Yang, Y. Containing pandemic influenza with antiviral agents. *Am J Epidemiol 159*, 7 (Apr 2004), 623–633.

[FLU160] Lupatkin, H. Influenza vaccine in the elderly and chronic obstructive pulmonary disease. *Curr Infect Dis Rep 7*, 3 (May 2005), 200–203.

[FLU92] Lyytikinen, O., Hoffmann, E., Timm, H., Schweiger, B., Witte, W., Vieth, U., Ammon, A., and Petersen, L. R. Influenza a outbreak among adolescents in a ski hostel. *Eur J Clin Microbiol Infect Dis 17*, 2 (Feb 1998), 128–130.

[FLU149] MacDonald, P., and Lyth, J. Incubation period of influenza. *The British Medical Journal 2* (1918), 488.

[FLU85] Madjid, M., Naghavi, M., Litovsky, S., and Casscells, S. W. Influenza and cardiovascular disease: a new opportunity for prevention and the need for further studies. *Circulation 108*, 22 (Dec 2003), 2730–2736.

[FLU110] McCain, J. Managed care (and everyone else) unprepared for the next killer flu. *Manag Care 14*, 2 (Feb 2005), 38, 41–2, 48–50.

[FLU44] McKendrick, A., and Morison, J. The determination of incubation periods from maritime statistics, with particular reference to the incubation period of influenza. *Indian J Med Res 7* (1919), 364–371.

[FLU292] Meissner, H. Reducing the Impact of Viral Respiratory Infections in Children. *The Pediatric Clinics of North America 52*, 3 (2005), 695–710.

[FLU131] Michigan Department of Community Health. Michigan Pandemic Influenza Plan, May 2007.

[FLU171] Ministry of Health, Trinidad and Tobago. Pandemic influenza preparedness and response plan. [Online accessed 17 April 2008], December 2005.

[FLU133] Mohamed, N. *Molecular diagnosis of common viral infectious diseases based on real-time PCR*. PhD thesis, Acta Universitatis Upsaliensis, 2006.

[FLU29] Moser, M. R., Bender, T. R., Margolis, H. S., Noble, G. R., Kendal, A. P., and Ritter, D. G. An outbreak of influenza aboard a commercial airliner. *Am J Epidemiol 110*, 1 (Jul 1979), 1–6.

[FLU123] Neumann, G., and Kawaoka, Y. Influenza epidemics. *Encyclopedia of Life Sciences* (2006).

[FLU106] Noakes, C. J., Beggs, C. B., Sleigh, P. A., and Kerr, K. G. Modelling the transmission of airborne infections in enclosed spaces. *Epidemiol Infect 134*, 5 (Oct 2006), 1082–1091.

[FLU121] Nyquist, A. Influenza virus mutation and transmission. *Managed Care (suppl.) 16*, 8 (August 2007), 6–9.

[FLU100] Olsen, G. W., Steinberg, M. E., and Ley, C. A. Worksite influenza immunization programs. insight into the implementation and cost-benefit. *AAOHN J 53*, 3 (Mar 2005), 105–110.

[FLU179] Oner, A. F., Bay, A., Arslan, S., Akdeniz, H., Sahin, H. A., Cesur, Y., Epcacan, S., Yilmaz, N., Deger, I., Kizilyildiz, B., Karsen, H., and Ceyhan, M. Avian influenza a (h5n1) infection in eastern turkey in 2006. *N Engl J Med 355*, 21 (Nov 2006), 2179–2185.

[FLU18] Ontario Ministry of Health and Long-Term Care. A Guide to the Control of Respiratory Infection Outbreaks in Long-Term Care Homes. [Online accessed 17 April 2008], October 2004.

[FLU291] Parsons, H. Report on the influenza epidemic of 1889–90. *London: Local Government Board, HMSO* (1891).

[FLU132] Porth, C. M. Alterations in respiratory function: infectious disorders and neoplasia. *Essentials in Pathophysiology* (2003).

[FLU129] Read, R., and Jennings, R. *Influenza: Human and Avian in Practice.* RSM Press, 2006.

[FLU16] Robinson, R. Influenza. *Health & Homeopathy* (2001).

[FLU119] Rodzinny, L. Prophylaxis and treatment of influenza: Guidelines of the college of family physicians in poland. `http://www.eswi.org/myUploadData/files/Flu_Guidelines_Polish_GPs.pdf` [Accessed 24 March 2008].

[FLU67] Schabas, R. Is the quarantine act relevant? *CMAJ 176*, 13 (Jun 2007), 1840–1842.

[FLU65] Sisley, R. *Epidemic influenza: notes on its origin and method of spread.* Longmans, Green, and Co., 1891.

[FLU30] Smith, A. P. Respiratory virus infections and performance. *Philos Trans R Soc Lond B Biol Sci 327*, 1241 (Apr 1990), 519–528.

[FLU122] Smith, N., Bresee, J., Shay, D., Uyeki, T., Cox, N., and Strikas, R. Prevention and Control of Influenza. *Recommendations of the Advisory Committee on immunization practices, MMWR 55* (2006), 1–42.

[FLU47] Smorodintseff, A., Tushinsky, A., and Korovin, A. Investigation of volunteers with the influenza virus. *Am J Med Sci 194* (1937), 159–70.

[FLU94] Sokos, D. R. Pharmacists' role in increasing pneumococcal and influenza vaccination. *Am J Health Syst Pharm 62*, 4 (Feb 2005), 367–377.

[FLU102] Spiteri, G., Farrugia, R., and Fenech, T. Influenza and the implications of a pandemic for malta. *Malta Med J 17*, 3 (October 2005), 6–14.

[FLU126] Steiner, M., Salvadori, M., Concepcion, K., Gimino, V., Kamat, D., Sibilia, S., and Fink, S. Index of Suspicion. *Pediatrics in Review 22*, 1 (2001), 22.

[FLU104] Taylor, C., Allen, A., Sumner, S., and Vought, M. Are you prepared to deal with a high-risk respiratory illness? *J Emerg Nurs 33*, 2 (Apr 2007), 110–118.

[FLU148] Togo, Y. A2 Hong Kong Influenza Incubation Period. *JAMA 209*, 12 (1969), 1911–1912.

[FLU101] Torok, M. Focus on field epidemiology. *North Carolina Center for Public Preparedness 1*, 6.

[FLU78] Tyring, S., Ed. *Mucosal immunology & virology.* Springer, 2006.

[FLU49] UK Ministry of Health. Pandemic of influenza 1918–19. *Reports on Public Health and Medical Subjects, His Majesty's Stationery Office*, 4 (1920).

[FLU120] University of California, Davis Health System. Influenza pandemic emergency response & business continuity plan, September 2006.

[FLU150] Van Zwalenburg, C. Is influenza transmissible during the period of incubation? *JAMA 71*, 21 (1918), 1764–65.

[FLU1] Viboud, C., Bolle, P.-Y., Cauchemez, S., Lavenu, A., Valleron, A.-J., Flahault, A., and Carrat, F. Risk factors of influenza transmission in households. *Br J Gen Pract 54*, 506 (Sep 2004), 684–689.

[FLU96] Weir, E. Coping with flu season. *CMAJ 162*, 6 (Mar 2000), 861.

[FLU63] Wesseling, G. Occasional review: influenza in copd: pathogenesis, prevention, and treatment. *Int J Chron Obstruct Pulmon Dis 2*, 1 (2007), 5–10.

[FLU209] Wilson, R., Alton, E., Rutman, A., Higgins, P., Al Nakib, W., Geddes, D. M., Tyrrell, D. A., and Cole, P. J. Upper respiratory tract viral infection and mucociliary clearance. *Eur J Respir Dis 70*, 5 (1987), 272–279.

[FLU236] Wilson, T., Gregg, D., King, D., Noah, D., Leigh Perkins, L., Swayne, D., and Inskeep, W. Agroterrorism, Biological Crimes, and Biowarfare Targeting Animal Agriculture. *Clin Lab Med 21*, 3 (2001), 549–591.

[FLU117] Wong, D., Blumberg, D., and Lowe, L. Guidelines for the use of antibiotics in acute upper respiratory tract infections. `http://www.aafp.org/afp/20060915/956.pdf` [Accessed 24 March 2008], 2006.

[FLU127] World Health Organization. Who consultation on priority public health interventions before and during an influenza pandemic, March 2004.

Measles References

[MEA16] Aaby, P. Patterns of exposure and severity of measles infection. copenhagen 1915-1925. *Ann. Epidemiol. 2*, 3 (1992), 257–262.

[MEA130] Aaby, P., Bukh, J., Lisse, I. M., and Smits, A. J. Severe measles in sunderland, 1885 - a european african comparison of causes of severe infection. *International Journal of Epidemiology 15*, 1 (1986), 101–107.

[MEA34] ABDULLAEV, S. [subjective sensations and functional disorders during the incubation period of measles.]. *Pediatriia. 40:86-7.* (1961), 86–87.

[MEA52] Aisenberg, A. C. Manifestations of immunologic unresponsiveness in hodgkin's disease. *Cancer Res 26*, 6 (1966), 1152–1164.

[MEA56] Akramuzzaman, S. M., Cutts, F. T., Hossain, M. J., Wahedi, O. K., Nahar, N., Islam, D., Shaha, N. C., and Mahalanabis, D. Measles vaccine effectiveness and risk factors for measles in dhaka, bangladesh. *Bulletin of the World Health Organization 80*, 10 (2002), 776–782.

[MEA29] Allerdist, H. Neurological complications following measles vaccination. *Dev. Biol. Stand. 43:259-64.* (1979), 259–264.

[MEA32] Allerdist, H., and Ehrengut, W. [measles and its complications in hamburg from 1960 to 1973 (author's transl)]. *Immun. Infekt. 4*, 3 (1976), 116–125.

[MEA7] Andreula, C. Cranial viral infections in the adult. *Eur. Radiol. 14 Suppl 3:E132-44.* (2004), E132–E144.

[MEA62] Babbott, F., and Gordon, J. E. Modern measles. *American Journal of Medical Sciences 228*, 3 (Sep. 1954), 334–361.

[MEA53] Bailey, N. T. J. Some problems in the statistical analysis of epidemic data. *Journal of the Royal Statistical Society. Series B (Methodological) 17*, 1 (1955), 35–68.

[MEA61] Barinaga, J. L., and Skolnik, P. R. Clinical presentation and diagnosis of measles.

[MEA5] Bedford, H. Measles and the importance of maintaining vaccination levels. *Nurs. Times. 100*, 26 (2004), 52–55.

[MEA37] Benenson, A., Ed. *Control of Communicable Diseases in Man*, 13th ed. 1980.

[MEA64] Bernstein, D. I., and Schiff, G. M. Measles. In *Infectious Diseases*, S. L. Gorbach, J. G. Bartlett, and N. R. Blacklow, Eds. W. B. Saunders, 1998.

[MEA135] Bhettay, E., Kipps, A., and Mcdonald, R. Early onset of subacute sclerosing panencephalitis. *Journal of Pediatrics 89*, 2 (1976), 271–272.

[MEA106] Black, F. L. In *Viral Infections of Humans, Epidemiology and Control*, A. S. Evans and R. A. Kaslow, Eds. 1997.

[MEA176] Blume, C. H. Erneute masern nach abge-schwachten masern. verlangerung der inkubationszeit. *Arch Kinderheilk 99*, (1) (1933), 49–50.

[MEA93] Boas, J. E. V. Bibliographie vom jahre 1884. *Archives of Dermatological Research 11*, 3 (1884), 553–622.

[MEA9] Butenandt, O., and Weiss, M. [measles. a viral illness with risk of permanent damage]. *MMW. Fortschr. Med. 141*, 37 (1999), 30–32.

[MEA77] Carson, M. A. Few young children had mild reactions to the measles, mumps, and rubella vaccine and older children had nearly no vaccine related reactions. *British Medical Journal 4*, 2 (2001), 42.

[MEA133] Casali, P., and Oldstone, M. B. A. Mechanisms of killing of measles virus-infected cells by human-lymphocytes - interferon associated and unassociated cell-mediated cyto-toxicity. *Cellular Immunology 70*, 2 (1982), 330–344.

[MEA] Cherry, J. D. In *Textbook of Pediatric Infectious Diseases.*

[MEA75] Cherry, J. D. *Textbook of Pediatric Infectious Diseases*, 3rd ed. W. B. Saunders, 1992.

[MEA110] Cherry, J. D. *Textbook of Pediatric Infectious Diseases*, 4th ed. W. B. Saunders, 1998.

[MEA63] Cherry, J. D. *Textbook of Pediatric Infectious Diseases*, 5th ed. W. B. Saunders, 2004.

[MEA50] Chin, J., Ed. *Control of Communicable Diseases Manual*, 17th ed. American Public Health Association, 2000.

[MEA40] Christensen, P. E., and Schmidt, H. An epidemic of measles in southern greenland, 1951: measles in virgin soil. iv. the significance of specific prophylaxis. *Acta Med. Scan. 145*, 2 (1953), 126–142.

[MEA36] Christensen, P. E., Schmidt, H., Bang, H. O., Andersen, V., Jordal, B., and Jensen, O. An epidemic of measles in southern greenland, 1951: measles in virgin soil. ii. the epidemic proper. *Acta Med. Scan. 144*, 6 (1953), 408–429.

[MEA39] Christensen, P. E., Schmidt, H., Bang, H. O., Andersen, V., Jordal, B., and Jensen, O. An epidemic of measles in southern greenland, 1951: measles in virgin soil. iii. measles and tuberculosis. *Acta Med. Scan. 144*, 6 (1953), 450–454.

[MEA38] Christensen, P. E., Schmidt, H., Jensen, O., Bang, H. O., Andersen, V., and Jordal, B. An epidemic of measles in southern greenland, 1951: I. measles in virgin soil. *Acta Med. Scan. 144*, 4 (1953), 313–322.

[MEA177] Chu, F. T., and Liu, S. C. The use of placental globulin extract in measles contact. an attempt to standardize the dosage. *Chinese Medical Journal 62*, 2 (1944), 111–118.

[MEA22] Cizman, M., Mozetic, M., Radescek-Rakar, R., Pleterski-Rigler, D., and Susec-Michieli, M. Aseptic meningitis after vaccination against measles and mumps. *Pediatr. Infect. Dis. J. 8*, 5 (1989), 302–308.

[MEA119] Czub, S., Lynch, W. P., Czub, M., and Portis, J. L. Kinetic-analysis of spongiform neurodegenerative disease induced by a highly virulent murine retrovirus. *Laboratory Investigation 70*, 5 (1994), 711–723.

[MEA83] da Silva Carneiro, S. C., Cestari, T., Allen, S. H., and e Silva, R. Viral exanthems in the tropics. *Clinics in Dermatology 25*, 2 (2007), 212–220.

[MEA100] DeBolt, C. A., and Sangeeta, R. Measles 2001: the roles of the epidemiologist and microbiologist in measles surveillance and outbreak control. Abstract, 2002.

[MEA4] Desopoulou, M., and Covanis, A. Subacute sclerosing panencephalitis after intrauterine infection. *Acta Paediatrica 93*, 9 (2004), 1251–1253.

[MEA174] Drinkwater, H. *Remarks upon the epidemic of measles prevalent in Sunderland. With notes upon 311 cases from middle Jan. to end of March 1885.* Edinburgh, 1885.

[MEA178] Dunkin, G., and Laidlaw, P. Studies in dog-distemper: Ii. experimental distemper in the dog. *Jour Comp Path & Therap 39*, (3) (1926), 213–221.

[MEA21] Dyken, P. R., Cunningham, S. C., and Ward, L. C. Changing character of subacute sclerosing panencephalitis in the united-states. *Pediatric Neurology 5*, 6 (1989), 339–341.

[MEA19] Enderle, J. D. A discrete-time communicable disease model with a stochastic contact rate for nonhomogeneous populations. *Biomed. Sci. Instrum. 27:77-88.* (1991), 77–88.

[MEA18] Farrington, C. P. Subacute sclerosing panencephalitis in england and wales - transient effects and risk estimates. *Statistics in Medicine 10*, 11 (1991), 1733–1744.

[MEA49] Fine, P. E. M. The interval between successive cases of an infectious disease. *American Journal of Epidemiology 158*, 11 (2003), 1039–1047.

[MEA99] for Disease Control, C., and Prevention. Update: measles among children adopted from china. *MMWR Morbidity and Mortality Weekly Report 53*, 21 (June 2004), 459.

[MEA31] Fratkin, J. D., and Smith, A. G. Slow virus infections. *Surv. Ophthalmol. 21*, 4 (1977), 356–365.

[MEA67] Gafafer, W. M. Peter ludwig panum's" observations on the contagium of measles". *Isis 24*, 1 (1935), 90–101.

[MEA101] Golding. Convincing adults to exercise. Electronic Citation, 2008.

[MEA73] Goodall, E. W. Measles with an illness at infection. *Clinical Journal 54* (1925), 69.

[MEA45] Goodall, E. W. Incubation period of measles. *British Medical Journal 1931* (1931), 73–74.

[MEA54] Gotlieb-Stematsky, T., Rannon, L., Vonsover, A., and Varsano, N. Stimulation of antibodies to epstein-barr virus (ebv) in acute viral infections. *Archives of Virology 57*, 3 (1978), 199–204.

[MEA2] Grais, R. F., Dubray, C., Gerstl, S., Guthmann, J. P., Djibo, A., Nargaye, K. D., Coker, J., Alberti, K. P., Cochet, A., Ihekweazu, C., Nathan, N., Payne, L., Porten, K., Sauvageot, D., Schimmer, B., Fermon, F., Burny, M. E., Hersh, B. S., and Guerin, P. J. Unacceptably high mortality related to measles epidemics in niger, nigeria, and chad. *PLoS Med. 4*, 1 (2007), e16.

[MEA139] Gray, M. J. Conception during rubella incubation period - report of case in which time of conception + exposure were precisely known. *Obstetrics and Gynecology 23*, 4 (1964), 526–&.

[MEA66] Greenwood, M. *Biometrika 36* (2008), 1–8.

[MEA179] Grist, N. R. The pathogenesis of measles: review of the literature and discussion of the problem. *Glasgow. Med. J. 31*, 12 (1950), 431–441.

[MEA170] Heymann, D. *Control of Infectious Diseases.* 2004.

[MEA107] Hirsch, A. *Handbook of geographical and historical pathology v. 3 1886*. New Sydenham Society, 1883.

[MEA180] Hurst, E. The neurotropic virus diseases. i. *Lancet. 229* (1935), 697–702.

[MEA181] Hurst, E. The neurotropic virus diseases. ii. *Lancet. 229* (1935), 758–762.

[MEA138] Imagawa, D. T. Propagation of rinderpest virus in suckling mice and its comparison to murine adapted strains of measles and distemper. *Archiv fur Die Gesamte Virusforschung 17*, 2 (1965), 203–&.

[MEA25] Katagiri, S., Ohizumi, A., Ohyama, S., and Homma, M. Follow-up study of type c influenza outbreak in a children's home. *Microbiol. Immunol. 31*, 4 (1987), 337–343.

[MEA108] Katz, S. L., Enders, J. F., Horsfall, F. L., and Tamm, I. Viral and rickettsial infections of man. 4th edit. Generic, 1965.

[MEA12] Keeling, M. J., and Grenfell, B. T. Effect of variability in infection period on the persistence and spatial spread of infectious diseases. *Mathematical Biosciences 147*, 2 (1998), 207–226.

[MEA97] Kronborg, D., Hansen, B., and Aaby, P. *Research report. 90/5. Variance component analysis of the incubation period for measles in the epidemic in Greenland, 1951*, vol. 90. Statistical Research Unit, University of Copenhagen, 1990.

[MEA17] Kronborg, D., Hansen, B., and Aaby, P. Analysis of the incubation period for measles in the epidemic in greenland in 1951 using a variance components model. *Stat. Med. 11*, 5 (1992), 579–590.

[MEA109] Krugman, S., Katz, S., Gershoti, A., and Wilfert, C. Infectious diseases of children. the cv mosby co. *Ltd. St. Louis, Toronto Princeton* (1985).

[MEA28] Kudo, Y., Sato, Y., Tsushima, N., Yokoyama, M., Matsumoto, K., and Takamatsu, H. [periodic thrombocytopenia occurring during the incubation period of measles]. *Rinsho Ketsueki. 24*, 10 (1983), 1355–1360.

[MEA23] Li, W. H., Ming, Z. L., Chen, Q., and Li, Y. Experimental studies on the prevention and treatment of chickenpox and herpes zoster with measles vaccine. *Chin Med. J. (Engl.). 102*, 5 (1989), 395–399.

[MEA88] Libansky, J. The investigations of the cellular type of immunity in patients with lymphoproliferative and myeloproliferative diseases. *International Journal of Cancer 4*, 3 (1969), 288–298.

[MEA20] Liebert, U. G., Schneider-Schaulies, S., Baczko, K., and Meulen ter, V. Antibody-induced restriction of viral gene expression in measles encephalitis in rats. *Journal of Virology 64*, 2 (1990), 706–713.

[MEA140] Liess, B., and Plowright, W. Studies on pathogenesis of rinderpest in experimental cattle .i. correlation of clinical signs viraemia + virus excretion by various routes. *Journal of Hygiene 62*, 1 (1964), 81–&.

[MEA11] Lisse, I., Samb, B., Whittle, H., Jensen, H., Soumare, M., Simondon, F., and Aaby, P. Acute and long-term changes in t-lymphocyte subsets in response to clinical and subclinical measles. a community study from rural senegal. *Scand. J. Infect. Dis. 30*, 1 (1998), 17–21.

[MEA80] Littmann, M., Nnockler, K., and Hallauer, J. Cluster of trichinellosis cases in mecklenburg-vorpommern, germany. *European Surveillance 11*, 20 (2006), 1.

[MEA42] London, W. P., and Yorke, J. A. Recurrent outbreaks of measles, chickenpox and mumps. i. seasonal variation in contact rates. *American Journal of Epidemiology 98*, 6 (1973), 453–468.

[MEA144] Meyer, M. B. An epidemiologic study of mumps - its spread in schools and families. *American Journal of Hygiene 75*, 2 (1962), 259–&.

[MEA147] Meyers, J. Study of the geographical and time progression of a measles epidemic in the mott haven health center district, new-york-city. *American Journal of Public Health 39*, 11 (1949), 1446–1450.

[MEA171] M.Knipe, D. *Fields Virology.* 2006.

[MEA96] Nandy, R., Handzel, T., Zaneidou, M., Biey, J., Coddy, R. Z., Perry, R., Strebel, P., and Cairns, L. Case-fatality rate during a measles outbreak in eastern niger in 2003. *Clin. Infect. Dis. 42*, 3 (2006), 322–328.

[MEA24] Narang, H. K. A chronological study of experimental scrapie in mice. *Virus Res. 9*, 4 (1988), 293–305.

[MEA69] Narita, M., Togashi, T., and Kikuta, H. Neonatal measles in hokkaido, japan. *The Pediatric Infectious Disease Journal 16*, 9 (1997), 908–909.

[MEA14] Nates, S. V., Rey, G. Y., Giordano, M. O., Zapata, M. T., Depetris, A., and Boshell, J. Modified seroneutralization assay for measles virus antibody detection. *Res. Virol. 145*, 1 (1994), 45–49.

[MEA72] Nelson, K. E. Invited commentary on hobservations on a mumps epidemic in a'virgin'population. *American Journal of Epidemiology 142*, 3 (2003), 231–232.

[MEA1] Nguyen, H. T., and Rohani, P. Noise, nonlinearity and seasonality: the epidemics of whooping cough revisited. *J. R. Soc. Interface. .* (2007).

[MEA115] Nielsen, N. M., Aaby, P., Wohlfahrt, J., Pedersen, J. B., Melbye, M., and Molbak, K. Intensive exposure as a risk factor for severe polio: A study of multiple family cases. *Scandinavian Journal of Infectious Diseases 33*, 4 (2001), 301–305.

[MEA13] Nihei, K. [subacute sclerosing panencephalitis (sspe)]. *Nippon Rinsho. 55*, 4 (1997), 908–915.

[MEA65] Nishiura, H. Early efforts in modeling the incubation period of infectious diseases with an acute course of illness. *Emerging Themes in Epidemiology 4* (2007), 2.

[MEA87] Norton, S. L. Population growth in colonial america: A study of ipswich, massachusetts. *Population Studies 25*, 3 (1971), 433–452.

[MEA104] of Pediatrics, A. A. Measles. In *Red Book: Report of the Committee on Infectious Diseases*, 24th ed. 1997, pp. 344–357.

[MEA26] Ohuchi, M., Ohuchi, R., and Mifune, K. Protective effect of measles virus inoculation on subacute sclerosing panencephalitis virus-infected mice. *Microbiol. Immunol. 30*, 3 (1986), 203–211.

[MEA27] Ohuchi, R., Ohuchi, M., and Mifune, K. Slow development of measles virus (edmonston strain) infection in the brain of nude mice. *Microbiol. Immunol. 28*, 7 (1984), 757–764.

[MEA10] Okada, H., Kobune, F., Sato, T. A., Kohama, T., Takeuchi, Y., Abe, T., Takayama, N., Tsuchiya, T., and Tashiro, M. Extensive lymphopenia due to apoptosis of uninfected lymphocytes in acute measles patients. *Archives of Virology 145*, 5 (2000), 905–920.

[MEA82] Oxman, M. N. Measles virus. *Clinical Virology,* (2002), 791–828.

[MEA141] Pampiglione, G. Prodromal phase of measles - some neurophysiological studies. *British Medical Journal 2*, 542 (1964), 1296–&.

[MEA79] Paneth, N. Island epidemics. Generic, 2002.

[MEA46] Panum, P. L. Observations made during the epidemic of measles on the faroe islands in the year 1846. delta omega society. *New York* (1940).

[MEA74] Partington, M. W., and Quinton, J. F. P. The preeruptive illness of measles. *Archives of Diseases of Children 34* (1959), 149–153.

[MEA3] Perucha, M., Ramalle-Gomara, E., Lezaun, M. E., Blanco, A., Quinones, C., Blasco, M., Gonzalez, M. A., Cuesta, C., Echevarria, J. E., Mosquera, M. M., and de, O. F. A measles outbreak in children under 15 months of age in la rioja, spain, 2005-2006. *European Surveillance 11*, 10 (2006), 267–270.

[MEA173] Pickering, L. K., and of Pediatrics Committee on Infectious Diseases, A. A. *Red Book: 2003 Report of the Committee on Infectious Diseases.* American Academy of Pediatrics, 2003.

[MEA105] Pickles, W. *Epidemiology in Country Practice.* The Williams and Wilkins company, 1939.

[MEA142] Plowright, W. Studies on pathogenesis of rinderpest in experimental cattle .2. proliferation of virus in different tissues following intranasal infection. *Journal of Hygiene 62*, 2 (1964), 257–&.

[MEA112] Poulsen, A., Cabral, F., Nielsen, J., Roth, A., Lisse, I. M., Vestergaard, B. F., and Aaby, P. Varicella zoster in guinea-bissau - intensity of exposure and severity of infection. *Pediatric Infectious Disease Journal 24*, 2 (2005), 102–107.

[MEA6] Ray, S. K., Mallik, S., Munsi, A. K., Mitra, S. P., Baur, B., and Kumar, S. Epidemiological study of measles in slum areas of kolkata. *Indian Journal of Pediatrics 71*, 7 (2004), 583–586.

[MEA8] Richardson, M., Elliman, D., Maguire, H., Simpson, J., and Nicoll, A. Evidence base of incubation periods, periods of infectiousness and exclusion policies for the control of communicable diseases in schools and preschools. *Pediatr. Infect. Dis. J. 20*, 4 (2001), 380–391.

[MEA81] Riddell, M. A., Lynch, P., Jin, L., and Chibo, D. Measles cases imported from europe to victoria, australia, march, 2006. *European Surveillance 11*, 20 (May 2006), 2.

[MEA30] Santoli, D., Trinchieri, G., and Lief, F. S. Cell-mediated cytotoxicity against virus-infected target cells in humans. i. characterization of the effector lymphocyte. *Journal of Immunology 121*, 2 (1978), 526–531.

[MEA43] Sartwell, P. E. The distribution of incubation periods of infectious disease. *American Journal of Hygiene 51*, 3 (1950), 310–318.

[MEA15] Sattenspiel, L., and Powell, C. Geographic spread of measles on the island of dominica, west indies. *Human Biology 65*, 1 (1993), 107–129.

[MEA90] Semba, R. D., and Bloem, M. W. *Nutrition and Health in Developing Countries.* Humana Press, 2001.

[MEA136] Sever, J. L., Jabbour, J. T., Ellenberg, J., and Beadle, E. Constant incubation period for subacute sclerosing panencephalitis - effect of measles vaccines. *Neurology 25*, 4 (1975), 364–364.

[MEA149] Simpson, R. E. H. The period of transmission in certain epidemic diseases - an observational method for its discovery. *Lancet 255*, NOV13 (1948), 755–760.

[MEA85] SIMPSON, R. E. H. Infectiousness of communicable diseases in the household (measles, chickenpox, and mumps). *Lancet. 20;2*, 12 (1952), 549–554.

[MEA91] Smith, E. *A Practical treatise on disease in children.* Wood, 1886.

[MEA48] Starr, S., and Berkovich, S. The depression of tuberculin reactivity during chickenpox. *Pediatrics 33*, 5 (1964), 769.

[MEA143] Starr, S., and Berkovich, S. Effects of measles gamma-globulin-modified measles + vaccine measles on tuberculin test. *New England Journal of Medicine 270*, 8 (1964), 386–&.

[MEA114] Stepanova, I., Racek, P., Havlova, M., Nevsimalova, S., and Jakoubkova, M. Subacute sclerosing panencephalitis. *Ceska A Slovenska Neurologie A Neurochirurgie 65*, 5 (2002), 359–364.

[MEA71] Stewart-Freedman, B., and Kovalsky, N. An ongoing outbreak of measles linked to the united kingdom in an ultra-orthodox jewish community in israel. *European Surveillance 12*, 9 (2007), 1.

[MEA44] Stillerman, M., and Thalhimer, W. Attack rate and incubation period of measles - significance of age and of conditions of exposure. *American Journal of Diseases of Children 67*, 1 (1944), 15–21.

[MEA156] Stocks, P. Incubation period of measles. *British Medical Journal 1931* (1931), 157–157.

[MEA76] Stocks, P., and Karn, M. A. A study of the epidemiology of measles. *Annals of Eugenics 3* (1928), 390.

[MEA103] Szego, E. "all changed, changed utterly": recollections of 40 years in general practice. *Med. J. Aust. 181*, 1 (2004), 21–22.

[MEA57] Tapiainen, T., and Heininger, U. Fever following immunization. *Expert Review of Vaccines 4*, 3 (2005), 419–427.

[MEA35] Toniolo, G. [exanthematous rash in incubation period of measles.]. *Clin. Pediatr. (Bologna.). 33*, 1 (1951), 43–50.

[MEA89] Uhlendorf, B. W. The intracellular systhesis and maturation of a viral precursor.

[MEA92] Verzeichnlss, E. Bibliographie vom jahre 1884. *Archives of Dermatological Research 16*, 1 (1884), 553–622.

[MEA47] Virtanen, M., Peltola, H., Paunio, M., and Heinonen, O. P. Day-to-day reactogenicity and the healthy vaccinee effect of measles-mumps-rubella vaccination. Generic, 2000.

[MEA33] Vivell, O. [active measles vaccination contraindicated during the incubation period?]. *Med. Klin. 67*, 39 (1972), 1263.

[MEA84] Watson, J. C., Hadler, S. C., Dykewicz, C. A., Reef, S., and Phillips, I. Measles, mumps and rubella–vaccine use and strategies for elimination of measles, rubella and congenital rubella syndrome and control of mumps: recommendations of the advisory committee on immunization practices. *MMWR Recomm Rep 47*, RR-8 (May 1998), 1–57.

[MEA55] Wilson, E. B., and Worcester, J. A second approximation to soper's epidemic curve. *Proceedings of the National Academy of Sciences of the United States of America 30*, 2 (February 1944), 37–44.

[MEA150] Wilson, E. B., and Worcester, J. The law of mass action in epidemiology. *Proceedings of the National Academy of Sciences of the United States of America 31*, 1 (1945), 24–34.

[MEA162] Wooley, R. E., Gilbert, J. P., and Whitehead, W. K. Survival of viruses in edible fermented waste materials. *American Journal of Veterinary Research 42* (1981), 87–90.

[MEA70] Zingher, A., and Mortimer, P. Convalescent whole blood, plasma and serum in the prophylaxis of measles: Jama, 12 april, 1926; 1180-1187. *Rev. Med. Virol. 15*, 6 (2005), 407–418.

[MEA102] Zwicky, F. On the physical characteristics of the perseus cluster of nebulae. *Proceedings of the National Academy of Sciences of the United States of America 28*, 9 (1942), 355–361.

Parainfluenza References

[PARA73] Aliyu, Z., Bordner, M., Henderson, D., and Barrett, J. Determinants and duration of viral shedding and transmission of human parainfluenza virus 3 infection among allogenic peripheral stem cell transplant patients. *Southern Medical Journal 98*, 10 (2005), S20–S21.

[PARA3] Cordle, R. *Tintinalli's Emergency Medicine: A Comprehensive Study Guide, 6th Ed.* McGraw-Hill, 2003, ch. Chapter 133. Upper Respiratory Emergencies.

[PARA2] Health Canada. A respiratory outbreak due to parainfluenza virus type 3 in a home for the aged - ontario. Tech. rep., Health Canada, 1996.

[PARA74] KAPIKIAN, A. Z., CHANOCK, R. M., REICHELDERFER, T. E., WARD, T. G., HUEBNER, R. J., and BELL, J. A. Inoculation of human volunteers with parainfluenza virus type 3. *JAMA 178* (Nov 1961), 537–541.

[PARA85] Karron, R. A., O'Brien, K. L., Froehlich, J. L., and Brown, V. A. Molecular epidemiology of a parainfluenza type 3 virus outbreak on a pediatric ward. *J Infect Dis 167*, 6 (Jun 1993), 1441–1445.

[PARA57] Muchmore, H. G., Parkinson, A. J., Humphries, J. E., Scott, E. N., McIntosh, D. A., Scott, L. V., Cooney, M. K., and Miles, J. A. Persistent parainfluenza virus shedding during isolation at the south pole. *Nature 289*, 5794 (Jan 1981), 187–189.

[PARA78] Murphy, B. *Infectious diseases.* Saunders, 1992, ch. Parainfluenza Viruses, pp. 1745–50.

[PARA79] Murphy, B. R., Richman, D. D., Chalhub, E. G., Uhlendorf, C. P., Baron, S., and Chanock, R. M. Failure of attenuated temperature-sensitive influenza a (h3n2) virus to induce heterologous interference in humans to parainfluenza type 1 virus. *Infect Immun 12*, 1 (Jul 1975), 62–68.

[PARA1] RENDTORFF, R. C., WALKER, L. C., and ROBERTS, A. N. A parainfluenza 3 virus outbreak in an orphanage nursery. *Am J Hyg 77* (Jan 1963), 82–97.

[PARA75] Smith, C. B., Purcell, R. H., Bellanti, J. A., and Chanock, R. M. Protective effect of antibody to parainfluenza type 1 virus. *N Engl J Med 275*, 21 (Nov 1966), 1145–1152.

[PARA76] TYRRELL, D. A., BYNOE, M. L., PETERSEN, K. B., SUTTON, R. N., and PEREIRA, M. S. Inoculation of human volunteers with parainfluenza viruses types 1 and 3 (ha 2 and ha 1). *Br Med J 2*, 5157 (Nov 1959), 909–911.

Rhinovirus References

[RHINO13] Andersen, I., Jensen, P. L., Reed, S. E., Craig, J. W., Proctor, D. F., and Adams, G. K. Induced rhinovirus infection under controlled exposure to sulfur dioxide. *Arch Environ Health 32*, 3 (1977), 120–125.

[RHINO6] Avila, P. C., Abisheganaden, J. A., Wong, H., Liu, J., Yagi, S., Schnurr, D., Kishiyama, J. L., and Boushey, H. A. Effects of allergic inflammation of the nasal mucosa on the severity of rhinovirus 16 cold. *J Allergy Clin Immunol 105*, 5 (May 2000), 923–932.

[RHINO2] Blomqvist, S. *Epidemiology of Human Rhinoviruses.* PhD thesis, University of Helsinki, Fiinland, 2004.

[RHINO16] Douglas, R. G., Cate, T. R., Gerone, P. J., and Couch, R. B. Quantitative rhinovirus shedding patterns in volunteers. *Am Rev Respir Dis 94*, 2 (Aug 1966), 159–167.

[RHINO29] Douglas, R. G., Rossen, R. D., Butler, W. T., and Couch, R. B. Rhinovirus neutralizing antibody in tears, parotid saliva, nasal secretions and serum. *J Immunol 99*, 2 (Aug 1967), 297–303.

[RHINO5] Drake, C. L., Roehrs, T. A., Royer, H., Koshorek, G., Turner, R. B., and Roth, T. Effects of an experimentally induced rhinovirus cold on sleep, performance, and daytime alertness. *Physiol Behav 71*, 1-2 (2000), 75–81.

[RHINO17] Fox, J. P., Cooney, M. K., and Hall, C. E. The seattle virus watch. v. epidemiologic observations of rhinovirus infections, 1965-1969, in families with young children. *Am J Epidemiol 101*, 2 (Feb 1975), 122–143.

[RHINO7] Harris, J. M., and Gwaltney, J. M. Incubation periods of experimental rhinovirus infection and illness. *Clin Infect Dis 23*, 6 (Dec 1996), 1287–1290.

[RHINO30] Kaslow, D. C. Biological feasibility of developing prophylactic vaccines for viral pathogens: incubation period as a critical parameter. *Hum Vaccin 3*, 1 (2007), 1–7.

[RHINO3] Lepore, M., Anolik, R., and Glick, M. *Burket's Oral Medicine, Diagnosis and Treatment*, 10th ed. BC Decker, 2003, ch. Diseases of the Respiratory Tract, pp. 341–362.

[RHINO14] Naclerio, R. M., Proud, D., Lichtenstein, L. M., Kagey-Sobotka, A., Hendley, J. O., Sorrentino, J., and Gwaltney, J. M. Kinins are generated during experimental rhinovirus colds. *J Infect Dis 157*, 1 (Jan 1988), 133–142.

[RHINO8] Tyrrell, D. A., Cohen, S., and Schlarb, J. E. Signs and symptoms in common colds. *Epidemiol Infect 111*, 1 (Aug 1993), 143–156.

[RHINO10] Wilson, R., Alton, E., Rutman, A., Higgins, P., Nakib, W. A., Geddes, D. M., Tyrrell, D. A., and Cole, P. J. Upper respiratory tract viral infection and mucociliary clearance. *Eur J Respir Dis 70*, 5 (May 1987), 272–279.

Respiratory Syncytial Virus References

[RHINO13] Andersen, I., Jensen, P. L., Reed, S. E., Craig, J. W., Proctor, D. F., and Adams, G. K. Induced rhinovirus infection under controlled exposure to sulfur dioxide. *Arch Environ Health 32*, 3 (1977), 120–125.

[RHINO6] Avila, P. C., Abisheganaden, J. A., Wong, H., Liu, J., Yagi, S., Schnurr, D., Kishiyama, J. L., and Boushey, H. A. Effects of allergic inflammation of the nasal mucosa on the severity of rhinovirus 16 cold. *J Allergy Clin Immunol 105*, 5 (May 2000), 923–932.

[RSV1] Berner, R., Schwoerer, F., Schumacher, R. F., Meder, M., and Forster, J. Community and nosocomially acquired respiratory syncytial virus infection in a german paediatric hospital from 1988 to 1999. *Eur J Pediatr 160*, 9 (Sep 2001), 541–547.

[RSV52] Black, C. P. Systematic review of the biology and medical management of respiratory syncytial virus infection. *Respir Care 48*, 3 (Mar 2003), 209–31; discussion 231–3.

[RHINO2] Blomqvist, S. *Epidemiology of Human Rhinoviruses.* PhD thesis, University of Helsinki, Fiinland, 2004.

[RSV35] Byrd, L. G., and Prince, G. A. Animal models of respiratory syncytial virus infection. *Clin Infect Dis 25*, 6 (Dec 1997), 1363–1368.

[RSV57] Chin-Yun, L. G., Glenn, F., Sheng-Tu, C., Yung-Tsung, H., and Huo-Yao, W. An outbreak of respiratory syncytial virus infection in an infant nursery. *Taiwan Yi Xue Hui Za Zhi 72*, 1 (Jan 1973), 39–46.

[RSV2] Darville, T., and Yamauchi, T. Respiratory syncytial virus. *Pediatr Rev 19*, 2 (Feb 1998), 55–61.

[RSV12] Domachowske, J. B., and Rosenberg, H. F. Respiratory syncytial virus infection: immune response, immunopathogenesis, and treatment. *Clin Microbiol Rev 12*, 2 (Apr 1999), 298–309.

[RHINO16] Douglas, R. G., Cate, T. R., Gerone, P. J., and Couch, R. B. Quantitative rhinovirus shedding patterns in volunteers. *Am Rev Respir Dis 94*, 2 (Aug 1966), 159–167.

[RHINO29] Douglas, R. G., Rossen, R. D., Butler, W. T., and Couch, R. B. Rhinovirus neutralizing antibody in tears, parotid saliva, nasal secretions and serum. *J Immunol 99*, 2 (Aug 1967), 297–303.

[RHINO5] Drake, C. L., Roehrs, T. A., Royer, H., Koshorek, G., Turner, R. B., and Roth, T. Effects of an experimentally induced rhinovirus cold on sleep, performance, and daytime alertness. *Physiol Behav 71*, 1-2 (2000), 75–81.

[RHINO17] Fox, J. P., Cooney, M. K., and Hall, C. E. The seattle virus watch. v. epidemiologic observations of rhinovirus infections, 1965-1969, in families with young children. *Am J Epidemiol 101*, 2 (Feb 1975), 122–143.

[RSV54] Gardner, P. S., Court, S. D., Brocklebank, J. T., Downham, M. A., and Weightman, D. Virus cross-infection in paediatric wards. *Br Med J 2*, 5866 (Jun 1973), 571–575.

[RSV32] Garzon, L. S., and Wiles, L. Management of respiratory syncytial virus with lower respiratory tract infection in infants and children. *AACN Clin Issues 13*, 3 (Aug 2002), 421–430.

[RSV46] Hall, C. B. Respiratory syncytial virus. *Isr J Med Sci 19*, 10 (Oct 1983), 889–891.

[RSV28] Hall, C. B. *Principles and Practice of Clinical Virology.* John Wiley & Sons Ltd, 2000, ch. Respiratory Syncytial Virus, pp. 293–306.

[RHINO7] Harris, J. M., and Gwaltney, J. M. Incubation periods of experimental rhinovirus infection and illness. *Clin Infect Dis 23*, 6 (Dec 1996), 1287–1290.

[RSV9] Hayden, F. G., and Ison, M. G. Respiratory viral infections: Infections caused by specific agents. online, 6 2006. accessed 1/31/2008.

[RSV43] Hertz, M. I., Englund, J. A., Snover, D., Bitterman, P. B., and McGlave, P. B. Respiratory syncytial virus-induced acute lung injury in adult patients with bone marrow transplants: a clinical approach and review of the literature. *Medicine (Baltimore) 68*, 5 (Sep 1989), 269–281.

[RSV58] Jackson, G. G., and Muldoon, R. L. Viruses causing common respiratory infections in man. 3. respiratory syncytial viruses and coronaviruses. *J Infect Dis 128*, 5 (Nov 1973), 674–702.

[RSV4] Jafri, H. S. Treatment of respiratory syncytial virus: antiviral therapies. *Pediatr Infect Dis J 22*, 2 Suppl (Feb 2003), S89–92; discussion S92–3.

[RSV53] JOHNSON, K. M., CHANOCK, R. M., RIFKIND, D., KRAVETZ, H. M., and KNIGHT, V. Respiratory syncytial virus. iv. correlation of virus shedding, serologic response, and illness in adult volunteers. *JAMA 176* (May 1961), 663–667.

[RSV44] KAPIKIAN, A. Z., BELL, J. A., MASTROTA, F. M., JOHNSON, K. M., HUEBNER, R. J., and CHANOCK, R. M. An outbreak of febrile illness and pneumonia associated with respiratory syncytial virus infection. *Am J Hyg 74* (Nov 1961), 234–248.

[RSV63] Karr, C., Lumley, T., Shepherd, K., Davis, R., Larson, T., Ritz, B., and Kaufman, J. A case-crossover study of wintertime ambient air pollution and infant bronchiolitis. *Environ Health Perspect 114*, 2 (Feb 2006), 277–281.

[RHINO30] Kaslow, D. C. Biological feasibility of developing prophylactic vaccines for viral pathogens: incubation period as a critical parameter. *Hum Vaccin 3*, 1 (2007), 1–7.

[RSV62] Kaslow, D. C. Biological feasibility of developing prophylactic vaccines for viral pathogens: incubation period as a critical parameter. *Hum Vaccin 3*, 1 (2007), 1–7.

[RSV48] Krasinski, K. Severe respiratory syncytial virus infection: clinical features, nosocomial acquisition and outcome. *Pediatr Infect Dis 4*, 3 (1985), 250–257.

[RSV47] KRAVETZ, H. M., KNIGHT, V., CHANOCK, R. M., MORRIS, J. A., JOHNSON, K. M., RIFKIND, D., and UTZ, J. P. Respiratory syncytial virus. iii. production of illness and clinical observations in adult volunteers. *JAMA 176* (May 1961), 657–663.

[RSV37] Lemen, R. J. Respiratory syncytial virus and bronchiolitis. *Zhonghua Min Guo Xiao Er Ke Yi Xue Hui Za Zhi 36*, 2 (1995), 78–85.

[RHINO3] Lepore, M., Anolik, R., and Glick, M. *Burket's Oral Medicine, Diagnosis and Treatment*, 10th ed. BC Decker, 2003, ch. Diseases of the Respiratory Tract, pp. 341–362.

[RSV29] Macartney, K. K., Gorelick, M. H., Manning, M. L., Hodinka, R. L., and Bell, L. M. Nosocomial respiratory syncytial virus infections: the cost-effectiveness and cost-benefit of infection control. *Pediatrics 106*, 3 (Sep 2000), 520–526.

[RSV18] Madge, P., Paton, J. Y., McColl, J. H., and Mackie, P. L. Prospective controlled study of four infection-control procedures to prevent nosocomial infection with respiratory syncytial virus. *Lancet 340*, 8827 (Oct 1992), 1079–1083.

[RSV34] McCarthy, C. A., and Hall, C. B. Respiratory syncytial virus: concerns and control. *Pediatr Rev 24*, 9 (Sep 2003), 301–309.

[RSV33] McNamara, P. S., Flanagan, B. F., Selby, A. M., Hart, C. A., and Smyth, R. L. Pro- and anti-inflammatory responses in respiratory syncytial virus bronchiolitis. *Eur Respir J 23*, 1 (Jan 2004), 106–112.

[RSV30] Mlinaric-Galinovic, G., Falsey, A. R., and Walsh, E. E. Respiratory syncytial virus infection in the elderly. *Eur J Clin Microbiol Infect Dis 15*, 10 (Oct 1996), 777–781.

[RHINO14] Naclerio, R. M., Proud, D., Lichtenstein, L. M., Kagey-Sobotka, A., Hendley, J. O., Sorrentino, J., and Gwaltney, J. M. Kinins are generated during experimental rhinovirus colds. *J Infect Dis 157*, 1 (Jan 1988), 133–142.

[RSV31] Piedra, P. A. Clinical experience with respiratory syncytial virus vaccines. *Pediatr Infect Dis J 22*, 2 Suppl (Feb 2003), S94–S99.

[RSV11] Polak, M. J. Respiratory syncytial virus (rsv): overview, treatment, and prevention strategies. *Newborn and Infant Nursing Reviews 4* (2004), 15–23.

[RSV3] Rohwedder, A., Keminer, O., Forster, J., Schneider, K., Schneider, E., and Werchau, H. Detection of respiratory syncytial virus rna in blood of neonates by polymerase chain reaction. *J Med Virol 54*, 4 (Apr 1998), 320–327.

[RSV45] Sterner, G., Wolontis, S., Bloth, B., and de Hevesy, G. Respiratory syncytial virus. an outbreak of acute respiratory illnesses in a home for infants. *Acta Paediatr Scand 55*, 3 (May 1966), 273–279.

[RHINO8] Tyrrell, D. A., Cohen, S., and Schlarb, J. E. Signs and symptoms in common colds. *Epidemiol Infect 111*, 1 (Aug 1993), 143–156.

[RSV16] Tyrrell, D. A., Cohen, S., and Schlarb, J. E. Signs and symptoms in common colds. *Epidemiol Infect 111*, 1 (Aug 1993), 143–156.

[RSV25] Valenti, W. M., Menegus, M. A., Hall, C. B., Pincus, P. H., and Douglas, R. G. Nosocomial viral infections: I. epidemiology and significance. *Infect Control 1*, 1 (1980), 33–37.

[RSV40] Weir, E., and Fisman, D. N. Respiratory syncytial virus: pervasive yet evasive. *CMAJ 170*, 2 (Jan 2004), 191.

[RSV5] Welliver, J. R., and Welliver, R. C. Bronchiolitis. *Pediatr Rev 14*, 4 (Apr 1993), 134–139.

[RHINO10] Wilson, R., Alton, E., Rutman, A., Higgins, P., Nakib, W. A., Geddes, D. M., Tyrrell, D. A., and Cole, P. J. Upper respiratory tract viral infection and mucociliary clearance. *Eur J Respir Dis 70*, 5 (May 1987), 272–279.

[RSV6] Wood, M. J. Viral infections in neutropenia–current problems and chemotherapeutic control. *J Antimicrob Chemother 41 Suppl D* (Jun 1998), 81–93.

[RSV17] Wright, S. A., and Bieluch, V. M. Selected nosocomial viral infections. *Heart Lung 22*, 2 (1993), 183–187.
